# Cell stress response impairs de novo NAD^+^ biosynthesis in the kidney

**DOI:** 10.1172/jci.insight.153019

**Published:** 2022-01-11

**Authors:** Yohan Bignon, Anna Rinaldi, Zahia Nadour, Virginie Poindessous, Ivan Nemazanyy, Olivia Lenoir, Baptiste Fohlen, Pierre Weill-Raynal, Alexandre Hertig, Alexandre Karras, Pierre Galichon, Maarten Naesens, Dany Anglicheau, Pietro E. Cippà, Nicolas Pallet

**Affiliations:** 1 University of Paris, INSERM UMRS1138, Cordeliers Research Center, Paris, France.; 2 Department of Medicine, Division of Nephrology, Ente Ospedaliero Cantonale, Lugano, Switzerland.; 3 Department of Biochemistry, Assistance Publique-Hôpitaux de Paris, Georges Pompidou European Hospital, Paris, France.; 4 PMM: The Metabolism-Metabolome Platform, Necker Federative Research Structure, INSERM US24/CNRS UMS3633, Paris, France.; 5 University of Paris, INSERM UMRS970, Paris Cardiovascular Research Center (PARCC), Paris, France.; 6 Department of Anesthesia, Surgical Resuscitation, Assistance Publique-Hôpitaux de Paris, Georges Pompidou European Hospital, Paris, France.; 7 Service of Nephrology, Foch Hospital, Suresnes, France.; 8 Service of Nephrology, Assistance Publique-Hôpitaux de Paris, Georges Pompidou European Hospital, Paris, France.; 9 INSERM UMRS1155, Common and Rare Kidney Diseases: From Molecular Mechanisms to Personalized Medicine, Sorbonne University, Paris, France.; 10 Department of Microbiology, Immunology and Transplantation, KU Leuven, Leuven, Belgium.; 11 Service of Nephrology and Transplantation, Assistance Publique-Hôpitaux de Paris, Necker Hospital, Paris, France.

**Keywords:** Metabolism, Nephrology, Bioenergetics, Cell stress, Diagnostics

## Abstract

The biosynthetic routes leading to de novo nicotinamide adenine dinucleotide (NAD^+^) production are involved in acute kidney injury (AKI), with a critical role for quinolinate phosphoribosyl transferase (QPRT), a bottleneck enzyme of de novo NAD^+^ biosynthesis. The molecular mechanisms determining reduced QPRT in AKI, and the role of impaired NAD^+^ biosynthesis in the progression to chronic kidney disease (CKD), are unknown. We demonstrate that a high urinary quinolinate-to-tryptophan ratio, an indirect indicator of impaired QPRT activity and reduced de novo NAD^+^ biosynthesis in the kidney, is a clinically applicable early marker of AKI after cardiac surgery and is predictive of progression to CKD in kidney transplant recipients. We also provide evidence that the endoplasmic reticulum (ER) stress response may impair de novo NAD^+^ biosynthesis by repressing QPRT transcription. In conclusion, NAD^+^ biosynthesis impairment is an early event in AKI embedded with the ER stress response, and persistent reduction of QPRT expression is associated with AKI to CKD progression. This finding may lead to identification of noninvasive metabolic biomarkers of kidney injury with prognostic and therapeutic implications.

## Introduction

Nicotinamide adenine dinucleotide (NAD^+^) is a cofactor involved in oxidation-reduction reactions and serves as an energy transfer intermediate in multiple metabolic pathways (1). NAD^+^ is an important cosubstrate for histone deacetylases (sirtuins) and poly(ADP-ribose) polymerases (PARPs), which regulate several aspects of cellular homeostasis (1). Cellular NAD^+^ reduction in aging and in association with several diseases contributes to overall fitness decline and to a lower resistance to cell stress. Conversely, increasing NAD^+^ content can prolong health and life span in experimental organisms (2, 3). Recent experimental and clinical data demonstrated a critical role for NAD^+^ homeostasis in acute kidney injury (AKI) (4–7). Proximal tubular cells (PTCs) are highly metabolically active, and their survival and function depend on the ability to couple their energetic needs to the regulation of energy generation, antioxidant responses, and mitochondrial biogenesis and quality control, all of which rely on cytosolic and mitochondrial NAD^+^ (5, 8). NAD^+^ is continuously degraded, and stable NAD^+^ cellular concentrations are maintained by constant new supply through the nicotinamide salvage pathway (nicotinamide is generated as a by-product of enzymatic activities) and biosynthetic pathways (Supplemental Figure 1; supplemental material available online with this article; https://doi.org/10.1172/jci.insight.153019DS1). NAD^+^ biosynthesis involves diverse dietary sources, including nicotinic acid, nicotinamide, nicotinamide riboside, and tryptophan. The de novo pathway converts dietary tryptophan to NAD^+^ in 8 steps, with the last catalyzed by quinolinate phosphoribosyltransferase (QPRT). Reduction in QPRT activity during AKI is considered critical in NAD^+^ biosynthetic impairment (6), but the molecular mechanisms determining reduced QPRT upon kidney injury and its role in long-term outcomes after AKI remain unclear. The clinical relevance of noninvasive monitoring of QPRT activity in patients with kidney disease is not established.

## Results

### Impaired NAD^+^ biosynthesis is a very early event in AKI.

**Figure 1:**
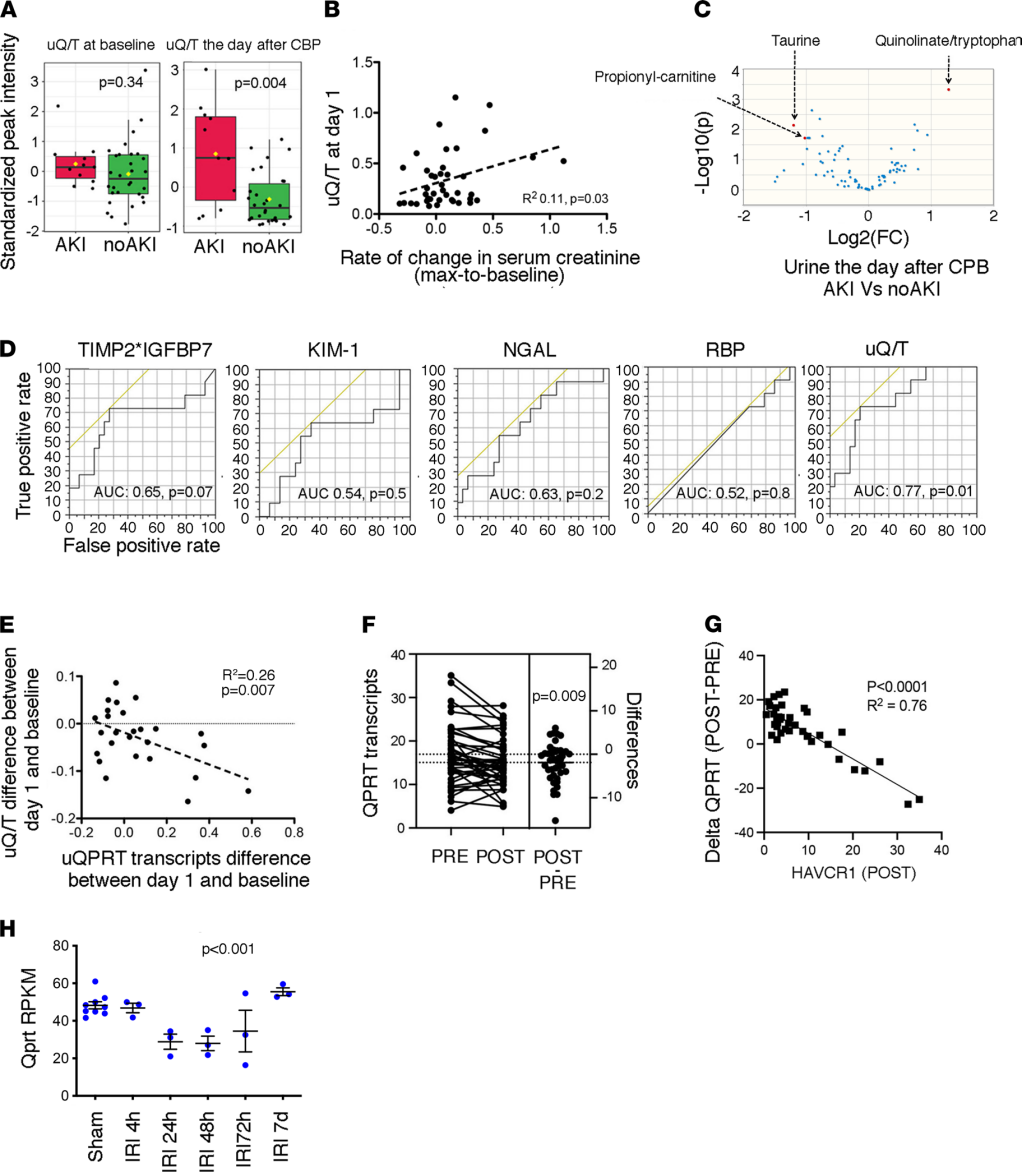
Impaired NAD^+^ biosynthesis is a very early event in AKI. (**A**) Distribution of quinolinate/tryptophan (uQ/T) levels measured in urine at baseline, and the day after cardiopulmonary bypass (CPB), according to the occurrence of AKI during the week after CPB. (**B**) Correlation between uQ/T levels measured in urine the day after CPB and the rate of variation in serum creatinine between the baseline and the maximum value in the week after CPB. *P* value was computed with Student’s *t* test. (**C**) Volcano plot comparing urinary metabolites of 41 patients collected 24 hours after CPB and who eventually developed AKI the week after surgery. (**D**) Receiver operating characteristic curves for the association between the concentrations of urinary tissue inhibitor of metalloproteinases-2 (TIMP-2) multiplied by insulin-like growth factor-binding protein 7 (IGFBP7), KIM-1, NGAL, retinol binding protein (RBP), and uQ/T collected the day after CPB and the occurrence of AKI the week after surgery. *P* values were computed with χ^2^ test, *n* = 41. (**E**) Increase of uQ/T levels between the day after CPB and baseline as a function of increase of urinary QPRT transcripts levels between the day after CPB and baseline. *P* value was computed with Student’s *t* test. (**F**) Expression of QPRT measured by RNA sequencing (RNA-Seq) of mRNA isolated from whole-kidney biopsies in a cohort of 42 KTRs before implantation (PRE) and shortly after the restoration of the blood flow (POST). *P* value was computed with Student’s *t* test. (**G**) Decrease of QPRT transcript levels as a function of the increase in HAVCR1 transcript levels measured by RNA-Seq of mRNA isolated from whole-kidney biopsies in a cohort of 42 KTRs shortly after the restoration of the blood flow (POST). *P* value was computed with Student’s *t* test. (**H**) Expression of Qprt transcripts measured by RNA-Seq of mRNA isolated from whole mouse kidneys examined at different time points following bilateral ischemia-reperfusion injury (IRI): (*n* = 3 to 4 mice per condition). *P* value was computed with 1-way ANOVA. RPKM, reads per kilobase per million mapped reads.

A previous study showed that quinolinate accumulates as a consequence of reduced QPRT activity and that urinary quinolate/tryptophan ratio (uQ/T) is elevated in AKI (6). Metabolic profiling of urine samples collected at baseline and the day after cardiopulmonary bypass (CPB) in 41 cardiac surgery patients (Supplemental Figure 3 for metabolomics data and Supplemental Table 2 for clinical data), including 11 patients (26%) developing AKI in the 7 days following CPB according to KDIGO criteria, showed that uQ/T was higher at day 1 after surgery in patients developing AKI, whereas at baseline, the levels were the same (Figure 1A), and severity of AKI correlated with uQ/T levels at day 1 (Figure 1B). uQ/T was one of a few urinary metabolic parameters significantly discriminating AKI at day 1 after surgery (but not at baseline) in this cohort (Figure 1C). uQ/T on day 1 after cardiac surgery predicted AKI with a higher accuracy than other AKI biomarkers, including kidney injury molecule 1 (KIM-1) or neutrophil gelatinase associated lipocalin (NGAL) (Figure 1D). In a multivariate analysis, uQ/T measured the day after CPB was an independent predictor of the occurrence of AKI (Supplemental Tables 3 and 4 for univariate and multivariate analyses). Urinary QPRT transcripts (urinary gene expression patterns reflect the dynamics of biopsy gene signatures; refs. 9–11) were negatively correlated with uQ/T levels, in line with the fact that elevated uQ/T is an indirect marker of reduced QPRT activity (Figure 1E). QPRT mRNA levels were significantly reduced in the first hours after ischemia-reperfusion injury (IRI) in protocol biopsies obtained in 42 kidney transplant recipients (KTRs) before and after reperfusion (12) (Figure 1F). QPRT transcripts levels were highly negatively correlated with HAVCR1 (the gene encoding KIM-1) transcripts (Figure 1G), suggesting that patients with more severe AKI in the posttransplantation biopsy had more pronounced QPRT reduction after reperfusion. Finally, in a mouse model of bilateral IRI (13), Qprt achieved the lowest level within 24 hours after IRI (Figure 1H). Thus, reduced QPRT transcription and activity is an early event associated with AKI. Indirectly determined by uQ/T in a clinical context, this is the first metabolic change to our knowledge predictive of AKI detectable by urinary metabolic profiling.

### QPRT reduction is associated with progression to chronic kidney disease.

**Figure 2:**
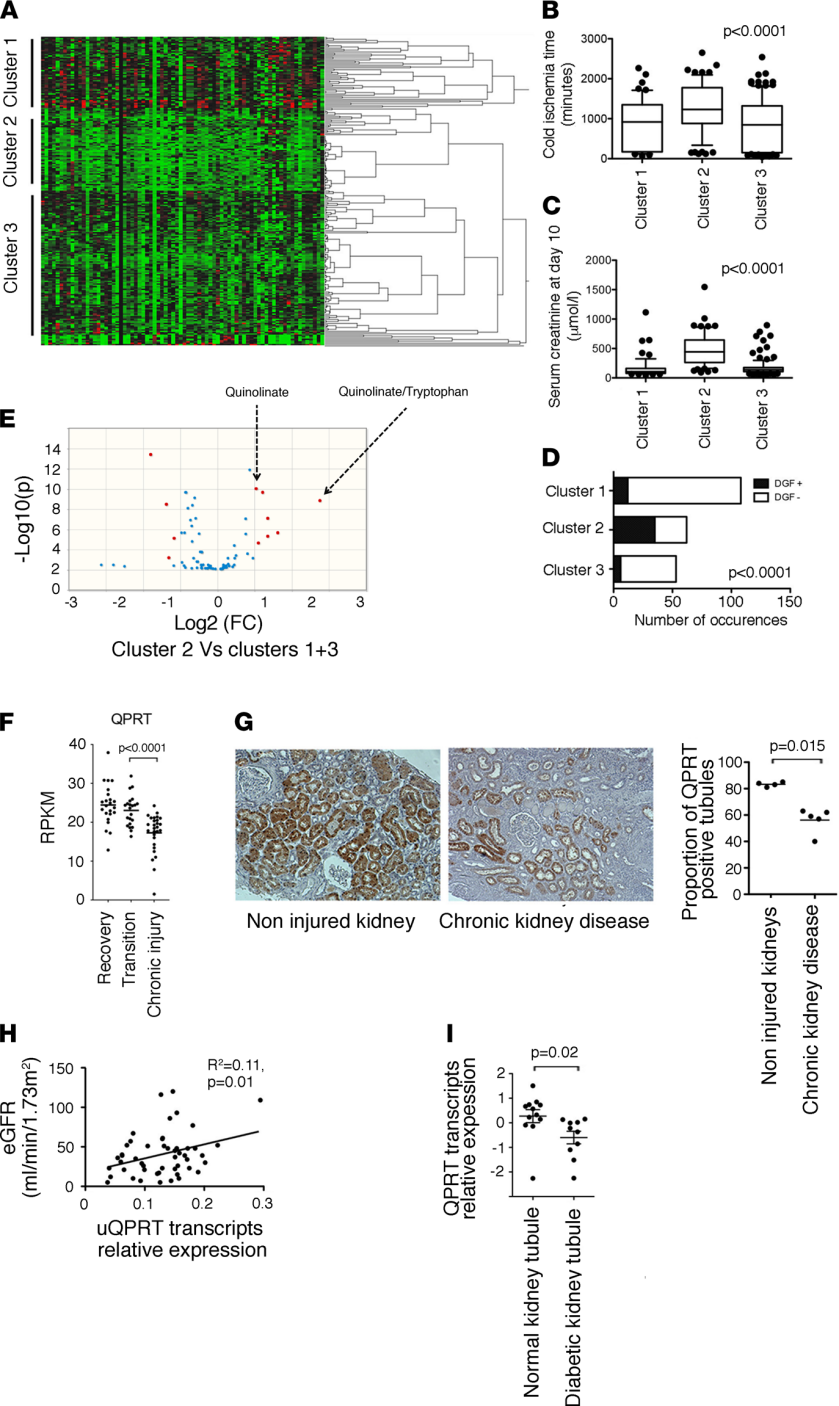
Persistent QPRT reduction is associated with progression to chronic kidney disease. (**A**) Hierarchical clustering of 237 urine samples collected 10 days after kidney transplantation. (**B**) Distribution of cold ischemia times, according to the clusters identified by hierarchical clustering in **A**. *P* value was computed using 1-way ANOVA. (**C**) Distribution of serum creatinine 10 days after transplantation, according to the clusters identified by hierarchical clustering in **A**. *P* value was computed using 1-way ANOVA. (**D**) Proportion of delayed graft function (DGF, need for a dialysis session during the 7 days after transplantation) events according to the clusters identified by hierarchical clustering in **A**. *P* value was computed using 1-way ANOVA. (**E**) Volcano plot comparing urinary metabolites of 273 patients collected 10 days after kidney transplantation in cluster 2 or clusters 1+3. Urinary quinolinate/tryptophan (uQ/T) remained significantly increased when using FDR-adjusted *P* values. (**F**) Expression of QPRT measured by RNA-Seq of mRNA isolated from whole kidneys in the group of 42 KTRs who recovered or progressed to fibrosis according to the computational model described (12), which identified 2 main transcriptional trajectories leading to kidney recovery or to sustained injury with associated fibrosis and renal dysfunction. *P* values were computed using Student’s *t* test. (**G**) Representative photomicrograph of human QPRT expression evaluated by immunohistochemistry in kidney tissue from an individual without chronic kidney disease (left) and with advanced chronic kidney disease (right). Original magnification, ×40. The distributions of QPRT-positive tubule sections corresponding to each condition (*n* = 4) were compared with Student’s *t* test. (**H**) Correlation between urinary QPRT transcript levels and estimated glomerular filtration rate in 55 patients examined for chronic kidney disease. *P* value was computed with Student’s *t* test. RPKM, reads per kilobase per million mapped reads. (**I**) Relative expression of QPRT transcripts in diabetic kidney disease tubulointerstitium samples (*n* = 10) compared with control samples (*n* = 12). *P* value was computed using Student’s *t* test. Data are from public repositories (National Center for Biotechnology Information Gene Expression Omnibus [NCBI GEO] accession GSE126805).

To investigate the long-term impact of the early reduction of de novo NAD^+^ biosynthesis associated with IRI, we performed a targeted urinary metabolic profiling in 237 KTRs 10 days after transplantation (Supplemental Table 5 for clinical data). Hierarchical clustering according to metabolic profiling identified 3 clusters (Figure 2A). Donor-related parameters, such as age, history of hypertension, renal function, or death from stroke (i.e., Extended Criteria Donor kidneys), were balanced among the clusters (Supplemental Figure 4, A and B), but cluster 2 was enriched with patients who underwent more severe ischemic injury, as indicated by longer cold ischemia time, higher serum creatinine the day of urine sampling, higher frequency of delayed graft function episodes (need for at least 1 dialysis session during the 7 days after transplantation), and higher number of dialysis sessions (Figure 2, B–D, and Supplemental Figure 4C). Among the urinary metabolites that best characterized cluster 2, quinolinate and uQ/T had the largest and most significant size effect (Figure 2E and Supplemental Table 6 for the list of metabolites). Critically, multivariate models integrating all variables associated with graft outcomes showed that high uQ/T 10 days after transplantation was an independent predictor of allograft function 3 and 12 months after transplantation (Table 1 and Table 2).

To investigate the role of QPRT along the transition from AKI to chronic kidney disease (CKD), we took advantage of the previously reported cohort of 42 KTRs followed by bulk RNA-Seq in protocol biopsies obtained 3 and 12 months after transplantation (12). A computational model to characterize the transcriptional transition from AKI to CKD has been developed, based on the principle that the progression to fibrosis is a transitional process from acute injury to fibrosis (12) (see Methods). According to this computational model, QPRT was progressively reduced along the transition to CKD (Figure 2F), a finding confirmed by immunohistochemistry in kidney biopsies obtained from patients with CKD (Figure 2G), by the correlation between renal function and QPRT transcript levels in urinary cell pellets in a cohort of 55 CKD patients (Figure 2H and Supplemental Table 7 for clinical data), and by a lower expression of QPRT transcripts in human diabetic renal tubular tissues (14) (Figure 2I). Thus, persistent QPRT reduction and impaired de novo NAD^+^ biosynthesis after AKI are features of the transition to chronic renal damage.

### ER stress reduces de novo NAD^+^ biosynthesis in the kidney.

**Figure 3:**
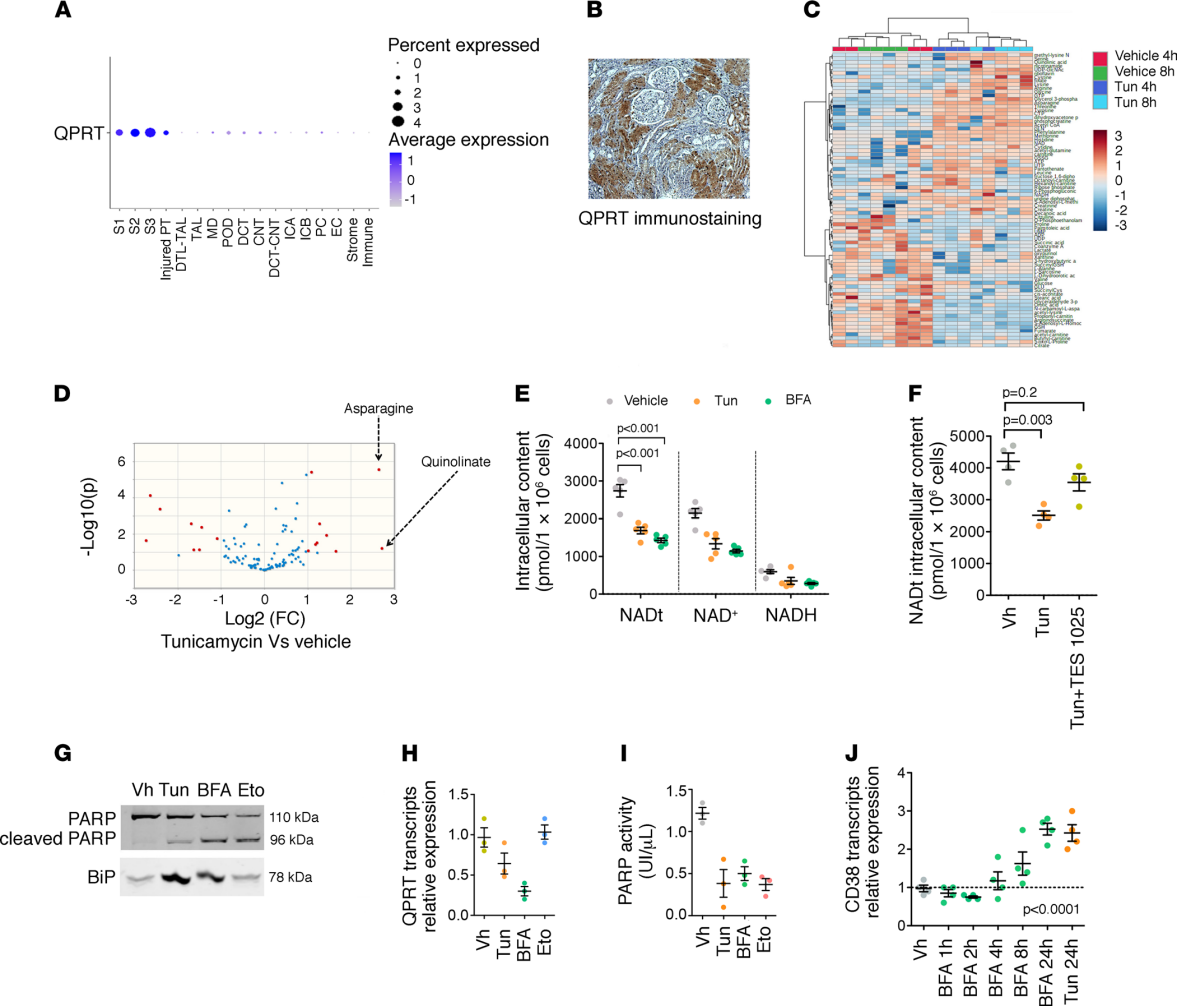
ER stress reduces de novo NAD^+^ biosynthesis in the kidney. (**A**) Qprt gene expression in the renal cell types identified by snRNA-Seq of 8 control kidney samples (25,896 cells). (**B**) Representative photomicrograph of human QPRT expression evaluated by immunohistochemistry in kidney. Original magnification, ×40. (**C**) Hierarchical clustering of HK2 cells incubated with 2.5 μg/mL of Tun or DMSO or vehicle for 4 hours and 8 hours (4 replicates). (**D**) Volcano plot comparing urinary metabolites of HK2 cells incubated with or without 2.5 μg/mL Tun for 8 hours. (**E**) Concentrations of total NAD concentrations, NAD^+^, and NADH, in HK2 cells incubated with 2.5 μg/mL Tun or 5 μg/mL BFA or DMSO for 24 hours (4–5 replicates). Bars represent mean ± SEM. *P* value was computed with Dunnett’s multiple-comparison test. (**F**) Concentrations of total NAD concentrations, NAD^+^, and NADH, in HK2 cells incubated with 2.5 μg/mL Tun alone with or without 100 μmol/L of TES1025 for 24 hours (*n* = 3–4 replicates). Bars represent mean ± SEM. *P* value was computed with Dunnett’s multiple-comparison test. (**G**) Immunoblot representing PARP, its cleaved fragment, and binding immunoglobulin protein (BiP) expression in HK2 cells 24 hours after incubation with DMSO, 2.5 μg/mL Tun, 5 μg/mL BFA, or 100 μM etoposide (Eto). The immunoblot shown is representative of 3 independent experiments. (**H**) Relative expression of QPRT measured by real-time quantitative polymerase chain reaction (RT-qPCR) in HK2 cells incubated with 2.5 μg/mL Tun, 5 μg/mL BFA, and 100 μM Eto or DMSO for 24 hours (3 replicates per condition). Bars represent mean ± SEM. (**I**) The PARylation activity of PARP in HK2 cells incubated with DMSO, 2.5 μg/mL Tun, 5 μg/mL BFA, or 100 μM Eto (3–4 replicates). Bars represent mean ± SEM. (**J**) Relative expression of CD38 measured by RT-qPCR in HK2 cells incubated with 2.5 μg/mL Tun or 5 μg/mL of BFA or DMSO for 24 hours (3–4 replicates per condition). Bars represent mean ± SEM. *P* value was computed with 1-way ANOVA.

We next investigated the potential mechanisms determining the reduction of NAD^+^ biosynthetic activity upon AKI. Among several factors involved in the early transcriptional response to AKI, we focused on the ER stress response, which is known to be activated in the early kidney injury response in mice (15, 16) and humans (17). We modeled the interaction between ER stress and NAD^+^ biosynthesis in PTCs by performing in vitro studies in the human kidney 2 (HK2) cell line, a proximal tubule–derived cell line expressing QPRT (Supplemental Figure 5). In line with this, analysis of mouse single-nucleus RNA-sequencing data sets indicated that Qprt was almost exclusively expressed in PTCs (Figure 3A), which is in line with transcriptomic and proteomic data in microdissected tubules in rats (18, 19) (Supplemental Figure 6, A and B) and supported by an immunohistochemistry study on normal human kidneys (Figure 3B).

In an unbiased approach, we induced ER stress with tunicamycin (Tun), an inhibitor of GlcNAc phosphotransferase that promotes ER stress and activates the unfolded protein response (UPR) (Supplemental Figure 7), and we performed metabolomics analysis in the cell lysate. ER stress profoundly reshaped the intracellular metabolic profile of HK2 cells (Figure 3C). Among the metabolites strongly enriched in response to ER stress, we found asparagine (ER stress induces asparagine synthase; refs. 20, 21), and quinolinate (Figure 3D and Supplemental Table 8A for the list of metabolites). Similar results were obtained after ER stress induction with brefeldin A (BFA) (Supplemental Figure 7), a lactone that inhibits protein transport from the ER to the Golgi complex (Supplemental Figure 8 and Supplemental Table 8B for the list of metabolites). ER stress induced by Tun or BFA reduced NAD^+^ intracellular contents (Figure 3E). This process was inhibited by TES1025 (Figure 3F), an inhibitor of the α-amino-β-carboxymuconate-ε-semialdehyde decarboxylase (ACMSD), indicating ER stress represses de novo NAD^+^ synthesis since ACMSD decreases the proportion of substrates able to undergo spontaneous cyclization into quinolinic acid (Supplemental Figure 1). Importantly, the decrease in NAD^+^ intracellular content was unlikely a consequence of accelerated consumption of NAD^+^ by PARPs, because ER stress was associated with PARP cleavage and defective PARylation activity (Figure 3, G–I). Note that etoposide, used as a positive control to promote apoptosis, did not induce ER stress and did not affect QPRT expression (Figure 3, G and H). We observed a slight increase in expression of the transcripts of the ectonuclease CD38 upon ER stress in HK2 cells (Figure 3J). CD38, an ectoenzyme capable of reducing extracellular NAD^+^ precursors’ availability (22), could theoretically participate in the decrease in NAD^+^ contents observed in ER-stressed cells, though the culture medium used (DMEM) contained high and likely saturating concentrations of nicotinamide (4 mg/L). These results suggest that ER stress may be involved in NAD^+^ biosynthetic impairment and that quinolinate accumulation is consistent with a reduction in the de novo biosynthetic pathway activity.

### QPRT expression is repressed in response to kidney injuries associated with ER stress.

**Figure 4:**
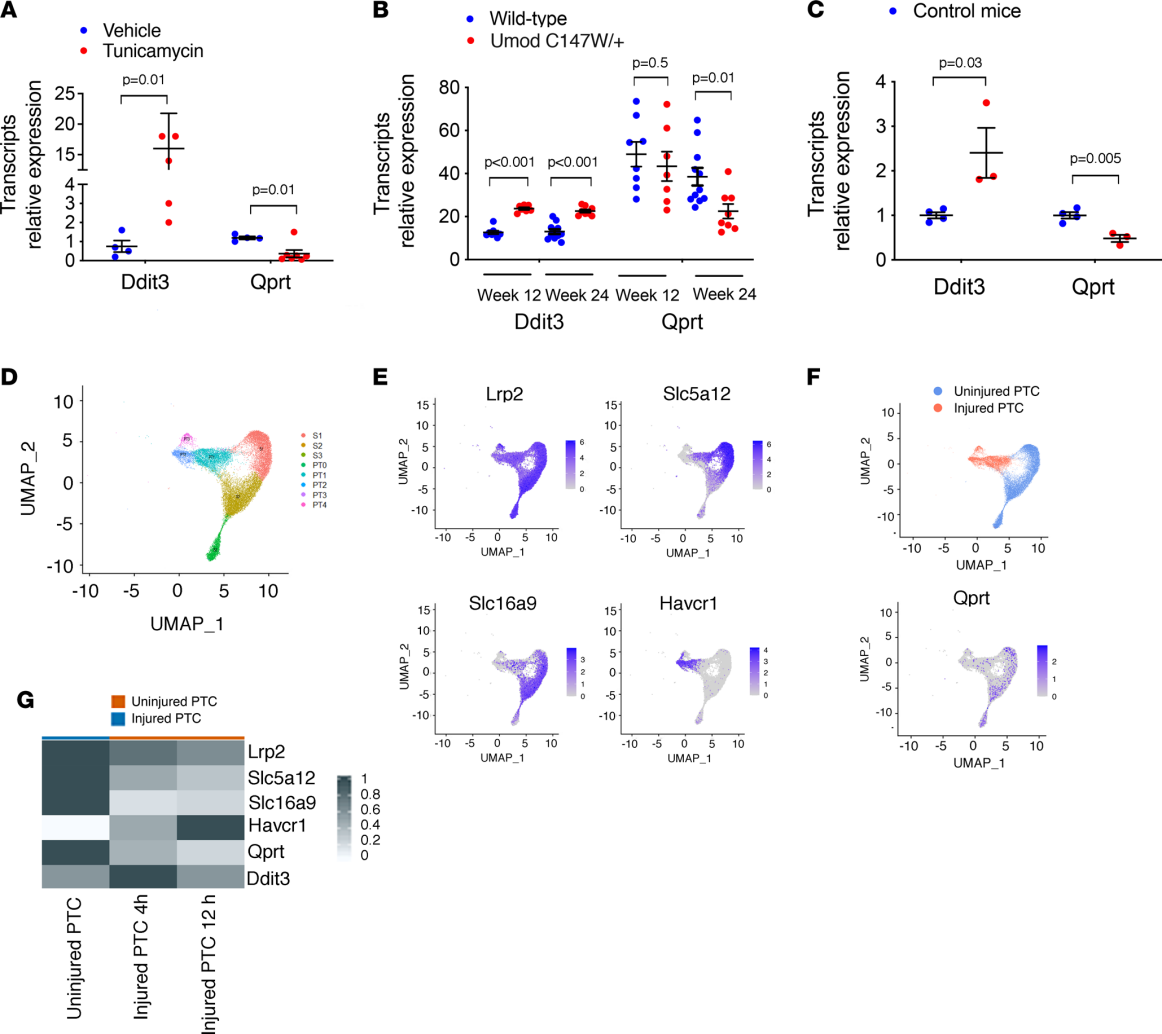
QPRT expression is repressed in response to kidney injuries associated with ER stress. (**A**) Expression of Ddit3 and Qprt transcripts by quantitative PCR (qPCR) in the kidney cortex of mice 48 hours after intraperitoneal injection of 1 mg/kg Tun or DMSO (*n* = 4 to 5 mice per condition). Bars represent mean ± SEM. *P* values were computed with Student’s *t* test. (**B**) Expression of Ddit3 and Qprt transcripts by RNA-Seq in whole kidneys of 12- and 24-week-old UmodC147W/+ mice and wild-type mice (5 to 10 mice per condition). Data are from public repositories (National Center for Biotechnology Information Gene Expression Omnibus [NCBI GEO] accession GSE102566). Bars represent mean ± SEM. *P* values were computed with Student’s *t* test. (**C**) Expression of Ddit3 and Qprt transcripts by qPCR in the kidney cortex of diabetic mice. Bars represent mean ± SEM. *P* values were computed with Student’s *t* test. (**D**) Uniform manifold approximation and projections (UMAPs) of 7 mouse IRI and 8 control kidney samples analyzed by snRNA-Seq (*n* = 19,926 cells) identify segments of the proximal tubule (S1, S2, S3) and new (injured) proximal tubule clusters (PT1, PT2, PT3). (**E**) UMAPs of 7 mouse IRI and 8 control kidney samples analyzed by snRNA-Seq (*n* = 19,926 cells) identify the expression of differentiation markers (Lrp2, Slc5a12, and Slc16a9) and injury marker (Havcr1) in segments of the proximal tubule (S1, S2, S3) and new proximal tubule clusters (PT1, PT2, PT3) (**F**) (Left) UMAPs of 7 mouse IRI and 8 control kidney samples analyzed by snRNA-Seq (*n* = 19,926 cells) highlighting uninjured (blue) and injured PTC (red) populations. (Right) UMAPs of 7 mouse IRI and 8 control kidney samples analyzed by snRNA-Seq (*n* = 19,926 cells) identify the differential expression of Qprt in injured and noninjured PTCs. (**G**) Expression of classical differentiation and injury markers over time after IRI in 7 mouse IRI and 8 control kidney samples analyzed by snRNA-Seq. Each column represents the average expression per cell state in control PTCs and injured PTCs at 4 hours and 12 hours. (*n* = 15 samples, *n* = 19,164 cells.)

A diminished expression of Qprt was found in several models of kidney injuries associated with ER stress — using DNA damage inducible transcript 3 (DDIT3), also known as C/EBP homologous protein, as a surrogate marker (23) — for instance, injection of Tun, a transgenic mouse model expressing mutant uromodulin (UmodC147W/+) that accumulates in the ER lumen, and streptozotocin-induced diabetic nephropathy (Figure 4, A–C). In addition, in the first hours after IRI in mice, single-nucleus RNA sequencing (snRNA-Seq) indicated that PTCs displayed a fundamental change of their transcriptional profile, with the expression of established injury markers (e.g., Havcr1), and the parallel loss of classical differentiation markers (e.g., Lrp2, encoding megalin), leading to the characterization of potentially novel PTC clusters (called PT1 to 3), which were referred to as “injured proximal tubule” (Figure 4, D and E; and Supplemental Figure 6, C–E) (8). PTCs in the early damaged state displayed low levels of Qprt (Figure 4F) associated with high levels of the ER stress marker Ddit3 (Figure 4G), indicating that the ER stress response is engaged in PTCs following IRI and that in this condition, Qprt expression is repressed.

**Figure 5:**
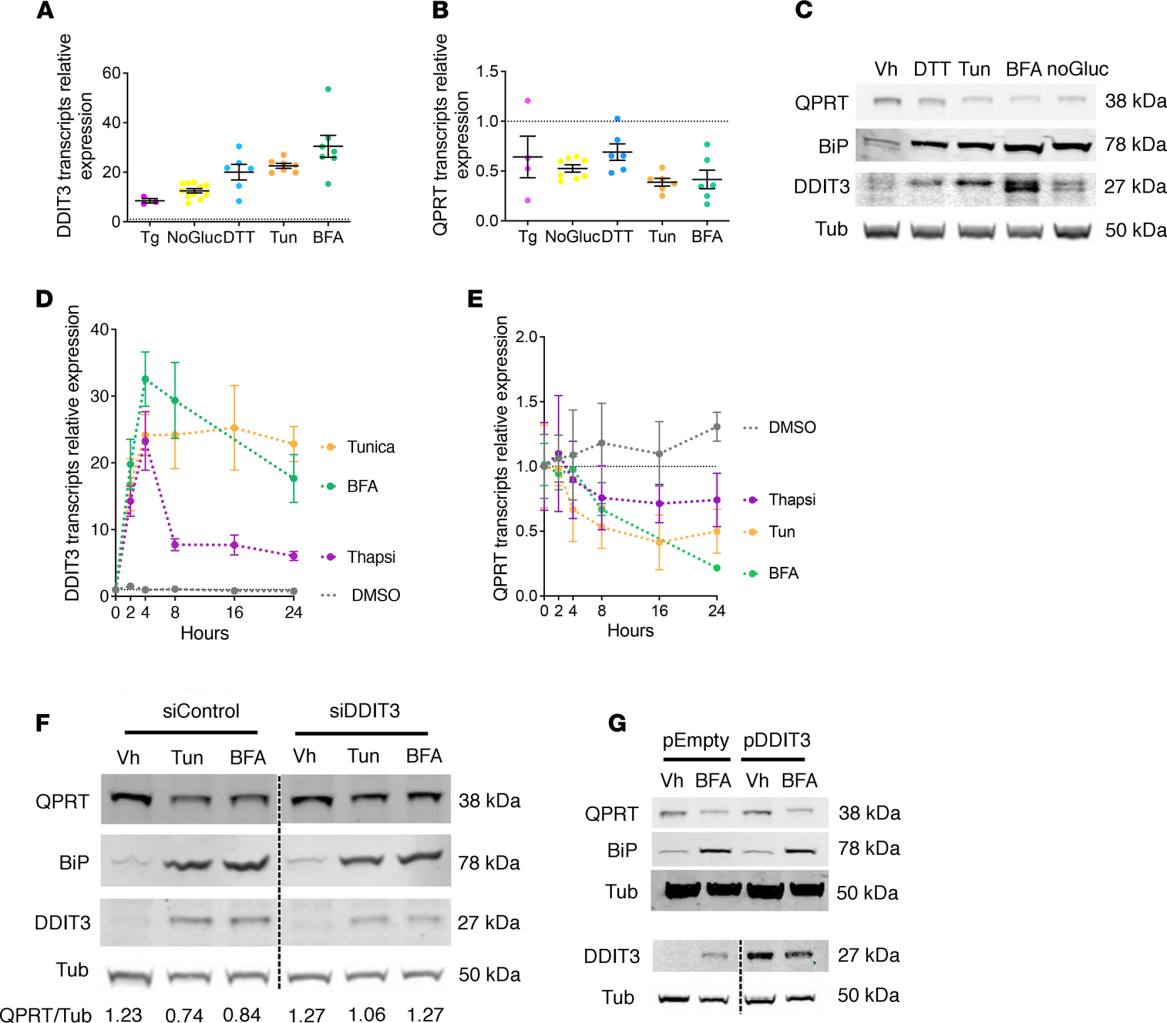
ER stress reduces QPRT expression in HK2 cells. (**A** and **B**) Scatter dot plots representing the relative expression of DDIT3 (**A**) and QPRT (**B**) transcripts measured by real-time quantitative polymerase chain reaction (RT-qPCR) in HK2 cells incubated 8 hours with 0.25 μM thapsigargin (Tg), with 1 μM dithiothreitol (DTT), with 2.5 μg/mL tunicamycin (Tun), with 5 μg/mL brefeldin A (BFA) or 48 hours in a glucose-deprived culture medium (No Gluc.), and compared with vehicle-treated (DMSO) cells (*n* = 6–8 replicates). The dashed line represents 1, the reference value of vehicle-treated cells. Bars represent mean ± SEM. (**C**) Immunoblot representing QPRT, binding immunoglobulin protein (BiP), DDIT3, and tubulin protein expression in HK2 cells 24 hours after incubation with DMSO (vehicle), 1 μM DTT, 2.5 μg/mL Tun, and 5 μg/mL BFA or 48 hours in a glucose-deprived culture medium (No Gluc.). The immunoblot shown is representative of 3 independent experiments. (**D** and **E**) Time course analysis of the relative expression of DDIT3 (**D**) and QPRT (**E**) transcripts measured by RT-qPCR in HK2 cells incubated with vehicle (DMSO), 0.25 μM Tg, 5 μg/mL BFA, or 2.5 μg/mL Tun for up to 24 hours (4 replicates). Bars represent mean ± SEM. (**F**) Immunoblot representing the expression of QPRT, BiP, DDIT3, and tubulin proteins in HK2 cells transfected with DDIT3 siRNA (siDDIT3) or with control siRNA and incubated with 2.5 μg/mL Tun, 5 μg/mL BFA, or DMSO (vehicle) for 24 hours. The immunoblot shown is representative of 2 independent experiments. (**G**) Immunoblot representing the expression of QPRT, BiP, DDIT3, and tubulin proteins in HK2 cells transfected with a pcDNA3.1 vector expressing DDIT3 or an empty vector and incubated with 5 μg/mL BFA or with DMSO (vehicle) for 24 hours. The immunoblot shown is representative of 2 independent experiments.

ER stress induced by Tun, BFA, thapsigargin (Tg, an inhibitor of the sarco/endoplasmic reticulum Ca^2+^-ATPase), dithiothreitol (DTT, a reducing agent), and glucose starvation activated the UPR and repressed QPRT levels in HK2 cells (Figure 5, A–C), whereas other key enzymes of the NAD^+^ biosynthesis and salvage pathways, including indoleamine 2,3-dioxygenase 1, ACMSD, nicotinic acid phosphoribosyl transferase, and nicotinamide phosphoribosyl transferase, were not affected by ER stress (Supplemental Figure 9). Time course analysis of QPRT transcript levels in HK2 cells after ER stress induction showed QPRT downregulation starting 4 hours after stimulation (Figure 5, D and E). The transcription factor DDIT3 has a repressive activity on numerous target genes (23–26), leaving the possibility that it could repress QPRT transcription. siRNA-mediated RNA interference against DDIT3 attenuated QPRT reduction upon ER stress (Figure 5F). The overexpression of DDIT3 in nonstressed cells had no impact on QPRT expression (Figure 5G), suggesting that additional factors embedded in the ER stress response are required for DDIT3 to repress QPRT. These findings were replicated in primary cultured PTCs (Supplemental Figure 10). Together, these results indicate that ER stress impairs QPRT expression and that DDIT3 may participate in QPRT repression.

## Discussion

A better understanding of the cellular and molecular processes activated in response to kidney injury, a better characterization of the structural determinants that foster progression from AKI toward CKD, and the identification of biomarkers of ongoing tissue injury in individuals during the early stages of the disease are required to improve patient care in nephrology. Our findings link the ER stress response to fundamental changes in PTC biology associated with AKI and AKI to CKD progression. Furthermore, this study provides a comprehensive characterization of noninvasive metabolic biomarkers of kidney disease that ultimately could be used to optimize therapies to slow disease progression.

We provide evidence for the first time to our knowledge that the ER stress response has a repressive role in NAD^+^ biosynthesis. The relationship between ER stress and cellular energetic metabolism is complex, and multiple pathways appear to be at work (27). Our results suggest a potentially novel metabolic role for the UPR in modulating NAD^+^ biosynthesis, possibly mediated by DDIT3. The consequences of these findings are multiple and raise several avenues for further investigation. The landscape of the biological impact of de novo NAD^+^ downregulation appears endless given the hundreds of redox reactions involving NAD^+^/NADH and dozens of reactions involving NAD^+^ consumption throughout the cell (28). Considering that NAD^+^ is a key modulator of mitochondrial homeostasis, a prime issue to be addressed is the way the UPR affects mitochondrial function and biogenesis. Indeed, the kidney is second only to the heart in the abundance of mitochondria, and the elevated energetic burden may render the kidney especially vulnerable to ischemia. This also underlines the need to better understand the heterogeneity of individual cellular stress responses and cell fate in the face of IRI. A subset of PTCs may fail to recover from acute stress and face an unresolved ER stress that may ultimately become deleterious and may prompt progression toward irreversible lesions. One could envision that early events that affect NAD^+^ contents in a proportion of PTCs imprint a metabolic memory in these cells, possibly through altered sirtuin activity and epigenetic reprogramming, which would endow them with a maladaptive phenotype, fueling fibrogenesis, inflammation, and progression to CKD if a critical threshold of PTC number is reached.

Our results suggest that DDIT3 could participate in the attenuation of QPRT transcription and contribute to the expanding body of data indicating that DDIT3 not only is an apoptotic regulator but also carries out nonapoptotic functions. It is clear that DDIT3 alone is not sufficient to inhibit metabolic gene expression because overexpressed DDIT3 was not associated with QPRT repression in the absence of ER stress. This observation suggests that ER stress signals are necessary to potentiate its repressive action. Our observations are reminiscent of what have been described for the suppression of genes encoding the transcriptional regulators of lipid metabolism such as PPARα, or SREBF1 by DDIT3 (25), raising the possibility that a regulatory program is engaged upon ER stress and could affect metabolic genes expression in a similar manner. In the case of NAD^+^ biosynthesis impairment upon ER stress, the precise mechanisms by which DDIT3 repress QPRT upon ER stress remain to be examined in detail. This transcriptional regulation of QPRT is not exclusive, and ER stress may affect QPRT enzyme activity through other mechanisms.

Our findings highlight the fact that a molecular reprogramming process occurring within a stressed cell in an injured tissue can be noninvasively detected and quantified and provide information regarding the magnitude of the damage. Implementing uQ/T as a noninvasive biomarker to improve prediction performance will provide a validated tool to increase the earliness and specificity of the diagnosis of kidney injury, which is a major prerequisite for successful intervention. It is anticipated that the earlier that therapy can be started, the greater the chances are to stop the pathological process. In agreement, the detection of increased uQ/T could lead to a better stratification of the risk of kidney disease progression. uQ/T is a new monitoring tool that could be routinely used in the follow-up of individuals with kidney disease. Individuals at high risk of disease progression will benefit from early nephroprotective strategies whose purpose is to increase intracellular NAD^+^ contents, and they would slow disease progression.

In conclusion, ER stress impairs de novo NAD^+^ biosynthesis, and the transcription factor DDIT3 may be involved in QPRT repression. Unresolved ER stress and QPRT downregulation constitute a signature of the transition from AKI to CKD. Finally, elevated uQ/T levels reflect the severity of tissue damage upon acute kidney allograft injury and are predictive of kidney outcomes.

## Methods

### Study populations

#### HEGP CPB cohort for urinary metabolome.

From February 17, 2017, to April 26, 2017, 42 patients undergoing scheduled cardiac surgery with CPB were enrolled. Detailed information on the cohort is available in Supplementary Table 2. The exclusion criteria were an estimated glomerular filtration rate (eGFR) < 30 mL/min/1.73 m^2^, an infusion of a radio contrast agent within the 24 hours before surgery, a preoperative left ventricular ejection fraction < 40%, age < 18 years, pregnancy, and the inability to provide written informed consent. AKI was diagnosed according to the KDIGO Clinical Practice Guideline for AKI criteria in measuring serum creatinine concentrations and urine output after the surgery. All medications acting on the renin-angiotensin system and all diuretics were stopped on the day before surgery. Lost blood was recovered using Cell Saver Elite (Haemonetics) and retransfused when possible. Vasotropic or inotropic agents, fluids, and transfusion products were administered at the discretion of the anesthesiologist based on clinical, echocardiographic, and biological findings. After surgery, all patients were transferred to the cardiovascular intensive care unit. Hemodynamic data were extracted from the Philips system using IXTREND software version 2.1.0 FW14 (Ixellence GmbH). Mean arterial pressure values were recorded every 1.25 seconds during the CPB procedure. Clinical data were prospectively extracted from the hospital’s electronic medical records. Urine samples were collected in Corning 50 mL conical tubes and centrifuged at 2000*g* for 20 minutes at 4°C within 4 hours of collection. Cell pellets were conserved in 300 μL of RLT buffer (Qiagen) and stored until mRNA extraction. Supernatants and cell pellets were stored at –80°C until analysis. All clinical data and samples were deidentified. Urine samples of 41 patients were technically adequate for further analysis. The urine samples used in this study have been part of a previously published analysis (29).

#### Leuven KTR cohort for kidney allograft RNA-Seq.

A total of 41 KTRs were enrolled at the University Hospitals of Leuven (12). In each case, protocol biopsy was performed at 4 time points: before implantation (PRE, kidney flushed and stored in ice), after reperfusion (POST, at the end of the surgical procedure), and 3 and 12 months after transplantation. Genome-wide gene expression profiling using RNA-Seq was performed in kidney allograft recipients as previously described (12). Based on RNA-Seq profiling of protocol biopsies, a computational model to characterize the transition from AKI to CKD has been developed, as previously described (12). Briefly, a pseudotime analysis including all 3-month and 12-month samples was performed to cover this transition. The pseudotime line separated into 2 branches: a branch with transcriptomes depicting the progression to fibrosis over time (called “transition” and “progression”) and an opposite branch with transcriptomes moving toward recovery (called “recovery”).

#### Necker KTR cohort for urinary metabolome.

All of the 405 consecutive patients who received a kidney transplant at the center from January 2010 to June 2012 were considered for this prospective, longitudinal, single-center cohort study. The reasons for exclusion were noninclusion criteria (*n* = 48), primary nonfunction/early graft loss (*n* = 12), other study with urine monitoring (*n* = 16), patients’ death within the first 6 months (*n* = 7), and early loss of follow-up (*n* = 22). Posttransplantation, urine was collected on day 10 for 237 out of 300 individuals initially included in the study. Glomerular filtration rate was measured using iohexol clearance calculation. Urine samples were centrifuged at 1000*g* for 10 minutes at 4°C within 4 hours of collection. The supernatant was collected after centrifugation and stored with protease inhibitors at –80°C. The urine samples used in this study have been part of a previously published analysis (30).

#### HEGP CKD cohort for urinary transcript monitoring.

Between November 2017 and February 2018, 55 consecutive individuals who were referred to the Service of Nephrology at the Georges Pompidou European Hospital for a kidney biopsy were evaluated for potential inclusion in the study. Indications for biopsy were eGFR < 60 mL/min and/or proteinuria > 0.5 g/L. Kidney biopsies were collected only for patient care. At biopsy, urine samples were collected for routine clinical chemistry analyses and stored at –80°C. Detailed information regarding the clinical, medical, demographic, biological, and histological status of the patients was collected using an information-based data warehouse.

### Urine analyses

#### ELISAs.

Urinary KIM-1 levels were quantified using the KIM-1 ELISA immunoassay (R&D Systems), NGAL using the Lipocalin-2/NGAL Quantikine ELISA Immunoassays (R&D Systems), and TIMP-2 and IGFBP7 using TIMP2 ELISA immunoassay (R&D Systems) and IGFBP7 ELISA immunoassay (MyBiosource), respectively, according to the manufacturer’s protocol. Multiplication of the 2 markers (TIMP-2*IGFBP7) was performed as previously described (31). Because the distribution of the values of urinary KIM-1, NGAL, RBP, and TIMP2*IGFBP7 concentrations was skewed, we performed analyses using log-transformed values to obtain a Gaussian distribution, but this did not change the results of the comparative study.

#### Clinical chemistry analyses.

The urine protein measurements were performed at the Clinical Chemistry Department of the Georges Pompidou European Hospital. The urinary levels of RBP were measured using a Siemens BN II nephelometer Analyzer II and kits from Siemens. Values considered normal were less than 0.5 mg/L.

#### Urine mRNA processing.

Urine samples from the HEGP CKD cohort were collected in Corning 50 mL conical tubes and centrifuged at 2000*g* for 20 minutes within 4 hours of collection. Cell pellets were conserved in 300 μL of RLT buffer (Qiagen) and stored until mRNA extraction. Supernatants and cell pellets were stored at –80°C until analysis. RNA was extracted from the pellets using the RNeasy Mini Kit (Qiagen) and reverse-transcribed into cDNAs using TaqMan Reverse Transcription Reagents (Applied Biosystems).

### Targeted metabolomics

HK2 cells were washed twice with ice-cold PBS, drained, snap-frozen in liquid nitrogen, and stored at –80°C until analyses. After addition of an extraction solution made of 50% methanol, 30% acetonitrile, and 20% water (32) (1 mL/1 × 10^6^ cells or 500 μL for 20 μL urine), the samples were vortexed for 5 minutes at 4°C, then centrifuged at 16,000*g* for 15 minutes at 4°C. The supernatants were collected and separated by liquid chromatography–mass spectrometry using SeQuant ZIC-pHilic column (MilliporeSigma). The aqueous mobile-phase solvent was 20 mM ammonium carbonate plus 0.1% ammonium hydroxide solution, and the organic mobile phase was acetonitrile. The metabolites were separated over a linear gradient from 80% organic to 80% aqueous for 15 minutes. The column temperature was 50°C and the flow rate was 200 μL/min. The metabolites were detected across a mass range of 75–1000 *m/z* using the Q-Exactive Plus mass spectrometer (Thermo Fisher Scientific) at a resolution of 35,000 (at 200 *m/z*) with electrospray ionization and polarity switching mode. Lock masses were used to ensure mass accuracy below 5 ppm. The peak areas of different metabolites were determined using Thermo Fisher Scientific TraceFinder software using the exact mass of the singly charged ion and known retention time on the HPLC column. Data analysis was performed in the MetaboAnalyst 4.0 software (33).

### Human kidney immunohistochemistry

Kidney biopsies were fixed in alcohol-formalin-acetic acid, dehydrated with ethanol and xylene, embedded in paraffin, and cut into 3 μm sections. Samples were then deparaffinized, rehydrated, and heated for 20 minutes at 97°C in citrate buffer. Endogenous peroxidase was inactivated by incubation for 10 minutes at room temperature in 0.3% H_2_O_2_. Sections were incubated with PBS containing 1:100 anti-QPRT (catalog orb317756, Biorbyt). Next, sections were incubated with anti-mouse antibody conjugated with horseradish peroxidase–labeled polymer (mixture of secondary antibodies of various species, Dako), then visualized with a peroxidase kit (Dako). Finally, the tissue sections were counterstained with hematoxylin.

### Animal experiments

#### Tun injection.

Twelve-week-old C57BL/6 background male mice (Charles River Laboratories) were intraperitoneally injected with Tun (MilliporeSigma, T7765) (1 mg/kg) or vehicle (DMSO) at day 0, and mice were sacrificed 2 days postinjection (*n* = 4–5 per condition). Total RNA was extracted from kidneys using the RNeasy Mini Kit according to the manufacturer’s protocol.

#### Induction of diabetes mellitus.

Twelve-week-old males of the 129/SvJ genetic background (Charles River Laboratories) were rendered diabetic by streptozotocin (MilliporeSigma, S-0130) (100 mg/kg in sodium citrate buffer, pH = 4.5) intraperitoneal injection on 2 consecutive days. Control mice received citrate buffer alone. Mice with fasting glycemia above 300 mg/dL were considered diabetic. Mice were euthanized 10 weeks after the induction of diabetes (*n* = 3–4 per condition). Total RNA was extracted from kidneys using the RNeasy Mini Kit (Qiagen) according to the manufacturer’s protocol.

#### UmodC147W/+ mouse.

A detailed description of the methods for the UmodC147W/+ mouse line of the C57BL/6J genetic background was previously reported (34). Public repositories pertaining to the transcriptome of mRNA isolated from whole-kidney tissue from mutant mice and littermate controls at multiple time points, including 12 and 24 weeks (GEO accession GSE102566), were analyzed for QPRT and DDIT3 expression.

#### Bilateral IRI.

This procedure has been detailed (13). A 21-minute warm IRI was performed, and cohorts of injured mice were examined at 2 and 4 hours and 1, and 2, and 3 days after IRI (*n* = 3–4). Warm renal IRI was performed on 10- to 12-week-old (25–28 g) C57BL/6CN male mice from Charles River Laboratories.

### Bulk RNA-Seq of kidneys after bilateral IRI

This procedure has been detailed (13).

### snRNA-Seq

snRNA-Seq data were obtained from an experimental model previously described (8). The experimental protocol is shown in Supplemental Figure 2. Seurat v3.2.0 in R v4 was used for analyses, including normalization, scaling, and clustering of nuclei. First, we analyzed each data set separately and excluded nuclei with fewer than 150 or more than 8000 genes detected. We also excluded nuclei with a relatively high percentage of unique molecular identifiers mapped to mitochondrial genes (>1) and ribosomal genes (>1, for normal kidney sample; and >2, all other samples). We performed curated doublet removal based on known lineage-specific markers. The samples from different data sets were integrated to avoid batch effect using Seurat standard workflow splitting by data set. Following ScaleData, RunPCA, FindNeighbours, and FindCluster at a resolution of 0.5 were performed. We focused our analysis on control and early time points after IRI (4 and 12 hours), resulting in 34,755 renal nuclei including 19,926 PTCs from 15 samples (8 controls and 7 after IRI). Cluster reassignment was performed based on manual review of lineage-specific marker expression. For data visualization, we used RunUMAP, FeaturePlot, and Dotplot from Seurat and dittoHeatmap from dittoSeq package.

### Cells

#### HK2 cell line.

Normal human renal epithelial cells of proximal origin (HK2) were purchased from ATCC/LGC Standards (lot number 60352186), then cultured according to a previously published method (35). HK2 is a cell line derived from primary PTCs. HK2 cells were cultured in DMEM containing 5 μg/mL insulin, 10 μg/mL human apotransferrin, 500 ng/mL hydrocortisone, 10 ng/mL epithelial growth factor, 6.5 ng/mL triiodothyronin, 5 ng/mL sodium selenite, 1% fetal calf serum (FCS), 25 IU/mL penicillin, 25 μg/mL streptomycin, and 10 mM HEPES buffer. These cells lines were mycoplasma free (Mycoalert Mycoplasma Detection Kit, Lonza). Tun, Tg, DTT, BFA, and etoposide were from MilliporeSigma.

#### Primary culture.

Human renal epithelial cells were harvested from human nephrectomy specimens removed for renal cell carcinoma, then isolated according to previously published methods, with minor modifications (36). Fragments of nonmalignant renal cortex were minced and digested with collagenase IV (250 IU/mL, MilliporeSigma) for 3 hours at 37°C. Cells were centrifuged at 3000*g* for 15 minutes at 4°C and the pellets washed 3 times with phosphate-buffered saline. Cells were then cultured in DMEM containing 5 μg/mL insulin, 10 μg/mL human apotransferrin, 500 ng/mL hydrocortisone, 10 ng/mL epithelial growth factor, 6.5 ng/mL triiodothyronin, 5 ng/mL sodium selenite, 1% FCS, 25 IU/mL penicillin, 25 μg/mL streptomycin, and 10 mM HEPES buffer. Cells were incubated at 37°C in 5% C0_2_ and 95% air. The characterization of our cellular model has been published previously (37), confirming the proximal descent of the vast majority of the cultured tubular epithelial cells. Experiments were not performed with cells beyond the third passage.

### RNA extraction and RT-qPCR

Total RNA was extracted using the RNeasy Mini Kit (Qiagen) according to the manufacturer’s protocol. Transcript expression levels were quantified through SYBR Green RT-qPCR using an ABI PRISM 7900 sequence detector system (Applied Biosystems). Vehicle-treated samples were used as controls, and the fold changes for each tested gene were normalized to the ribosomal protein L13A housekeeping gene. The relative expression levels were calculated using the 2^-ΔΔCT^ method (38). By definition, the expression level of a given gene in a control sample, using the 2^-ΔΔCT^ method to calculate relative expression levels, is 1. Primer sequences are listed in Supplemental Table 1.

### Protein extraction and immunoblotting

Cells were washed in PBS and incubated for 30 minutes at 4°C and in mPER lysis buffer (Thermo Fisher Scientific) with protease (Halt Protease Inhibitor Cocktail 100X, Thermo Fisher Scientific) and phosphatase inhibitors (Halt Phosphatase Inhibitor Cocktail 100X, Thermo Fisher Scientific). Extracts were centrifuged at 14,000*g* for 15 minutes at 4°C. Protein concentrations in the supernatant were measured by using a Pierce BCA Protein Assay Kit (Thermo Fisher Scientific) and Tecan Safire plate reader. Protein extracts (25 μg) were resolved by 4%–12% SDS-PAGE (Invitrogen) and transferred to nitrocellulose membranes (iBlot, Invitrogen). Membranes were blocked with SEABLOCK blocking buffer (Thermo Fisher Scientific) for 1 hour at room temperature and then incubated overnight at 4°C with primary antibody diluted in blocking buffer. Primary antibodies were anti-QPRT (catalog HPA011887, MilliporeSigma), anti-DDIT3 (catalog L63F7, Cell Signaling Technology), anti-PARP (catalog 95424, Cell Signaling Technology), anti-BiP (GRP78) (catalog sc-1050, Santa Cruz Biotechnology), anti-PERK (catalog C33E10, Cell Signaling Technology), anti-eiF2α (catalog 9722, Cell Signaling Technology), anti–phospho-eiF2α (catalog 9721, Cell Signaling Technology), and anti-Tubulin (catalog T9026, MilliporeSigma). After washings in PBS-Tween buffer, membranes were incubated with secondary antibodies coupled to IRDye fluorophores: goat anti–rabbit IgG (H+L) Alexa Fluor Plus 800 (catalog A32735, Thermo Fisher Scientific) and goat anti–mouse IgG Alexa Fluor 680 (catalog A-21057, Thermo Fisher Scientific). Infrared signal of membranes was revealed using an Odyssey detection system (Li-Cor Biosciences).

### siRNA transfections

The transient inactivation of DDIT3 was achieved using small interfering synthetic RNAs (siRNAs) designed by and obtained from Qiagen and transfected using HiPerFect (Qiagen) according to the manufacturer’s protocol. Two different siRNAs directed against the same target were transfected: Hs_DDIT3_1 FlexiTube siRNA (ref. SI00059528) andHs_DDIT3_3 FlexiTube siRNA (ref. SI00059542). AllStars Negative Control siRNA (5′-AACGAUGACACGAACACACTT-3′) has no homology to any known mammalian gene, and validation was performed using Affymetrix GeneChip arrays and a variety of cell-based assays to ensure minimal nonspecific effects on gene expression and phenotype. Cells were incubated with siRNA for 24 hours before conducting the experiments.

### Expression vectors

Cells were cultured at 37°C in 5% CO_2_ and were studied while subconfluent. Transient transfection of the gene DDIT3_OHu16873C_pcDNA3.1(+) subcloned by HindIII/BamHI in a pcDNA3.1 vector (catalog OHu16873C, GenScript) was performed using Lipofectamine 2000 (Invitrogen) according to manufacturer instructions. pcDNA3.1 vector was used as a control. After 36 hours of transfection, cells were harvested for mRNA and protein preparation.

### NAD measurement

The levels of NAD^+^ and intracellular NADH were measured by a colorimetric enzymatic test (BioVision, K337-100) according to the manufacturer’s protocol. The cells of a determined number of wells containing the 2 × 10^5^ cells required for each test were washed with ice-cold PBS, lysed with 400 μL of extraction buffer by 2 cycles of freeze-thaw (freezing 10 minutes at –80°C, thawing 10 minutes on dry ice), harvested in tubes, vortexed for 10 seconds, and centrifuged at 19,000*g* for 5 minutes at 4°C. Concentrations of NAD^+^ or NADH in cell lysates were measured at 450 nm against a calibration range with an Infinite 200 Plate Reader (Tecan). The levels of the dinucleotides were expressed per million cells.

### PARP activity

The levels of PARP activity were measured by ELISA (PARP/Apoptosis Colorimetric Assay Kit, catalog4684-096-K, R&D Systems) according to the manufacturer’s protocol. A total of 5 × 10^4^ cells/200 μL fresh medium/well in a 96-well, flat-bottom plate were incubated 24 hours for ER stressors or 10 mM etoposide. Samples of 25 μL containing 200 μg proteins were evaluated in triplicate. PARP activity in samples was evaluated by semiquantitatively detecting PAR deposited onto immobilized histone proteins in a 96-well format. An anti-PAR monoclonal antibody, goat anti-mouse IgG-HRP conjugate (both from the kit), and HRP substrate were used to generate a colorimetric signal (450 nm). Absorbance correlates with PARP activity.

### Data availability

RNA-Seq data for human kidney transplant biopsies are available at GEO (GSE126805). Transcriptomic data for human diabetic kidneys are available at GEO (GSE30122). RNA-Seq data for mouse IRI are available at GEO (GSE52004). RNA-Seq data for UmodC147W/+ kidneys and UMOD-expressing epithelium are available at GEO (GSE1102566). RNA-Seq and proteomic data for rat microdissected tubules are available at GEO (GSE56743 and PXD16958). RNA-Seq data for renal cell lines are available at GEO (GSE135441, GSE111837, and GSE99701). snRNA-Seq data are available at GEO (GSE151167, GSE139107, and GSE163863). Raw data of metabolomics analyses and clinical data are available from the corresponding authors upon reasonable request.

### Statistics

Graphs were generated using GraphPad Prism 7 (GraphPad Software, Inc.). Statistical analyses were performed with JMP.10 software (SAS Institute Inc.). Student’s *t* tests were 2 tailed and 1-way ANOVA was performed when indicated. A *P* value less than 0.05 was considered significant.

### Study approval

#### HEGP CPB cohort.

This single-center, prospective, pilot study was approved by the Comité de Protection des Personnes Sud Est III (CPP Sud Est III 2016-072 B), Bron, France, on February 7, 2017, and registered under EudraCT 2016-A01871-50. All patients provided written informed consent for study participation and for the biological analysis before inclusion.

#### Leuven KTR cohort for kidney allograft RNA-Seq.

Patients were enrolled at the University Hospitals of Leuven (12). Participants provided written informed consents, and the Ethical Review Board of the University Hospitals of Leuven (S53364 and S59572), Leuven, Belgium, approved this procedure and studies of humans.

#### Necker KTR cohort.

This study was approved by the Ethics Committee of Ile-de-France XI (approval 13016), Paris, France, and all the participating patients provided written informed consent.

#### HEGP CKD cohort.

Analyses were performed on deidentified patient samples. The patients were informed about the study and did not object to the use of their clinical and biological data collected as part of their clinical care exclusively. Data management complied with the French reference methodologies guidelines MR004 published by the commission nationale informatique et liberté, which apply to research carried out as part of clinical care (Comité de Recherche non CPP [institutional review board], CERAPHP Centre, Paris, France).

#### Animal experiments.

Experiments were conducted according to French veterinary guidelines and those formulated by the European Commission for experimental animal use (L358–86/609EEC) and/or to the *Guide for the Care and Use of Laboratory Animals* as published by the US NIH (National Academies Press, 2011). Animals were fed ad libitum, had free access to water, and were housed at constant ambient temperature in a 12-hour light/12-hour dark cycle.

## Author contributions

NP conceived and designed the project. NP, YB, AR, ZN, and VP performed experiments. NP analyzed CPB data sets. IN performed metabolomic analyses. AH and PG validated data interpretation. OL provided diabetic mouse kidneys. BF and PWR generated the CPB database urine biocollection. AK generated HEGP CKD data sets. MN and PEC provided RNA-Seq data from kidney allograft recipients and PEC performed analyses. DA generated Necker’s KTR data sets and urine biocollection. PEC and AR conducted snRNA-Seq experiments and analysis. NP and PEC wrote the manuscript. First authorship order was assigned according to the relative amount of data generated in the project in agreement with the 3 coauthors (YB, AR, and ZN).

## Supplementary Material

Supplemental data

## Figures and Tables

**Table 1:**
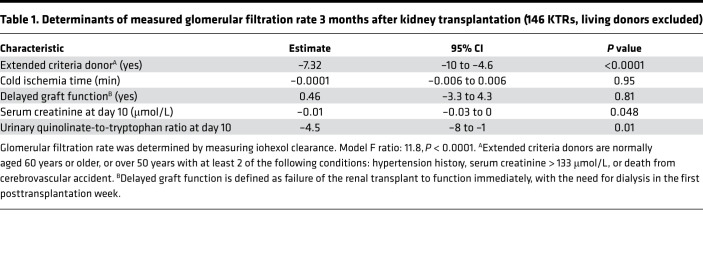
Determinants of measured glomerular filtration rate 3 months after kidney transplantation (146 KTRs, living donors excluded)

**Table 2:**
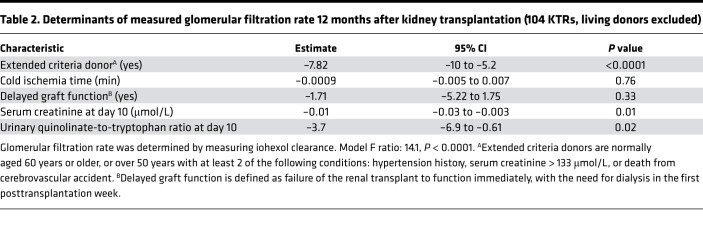
Determinants of measured glomerular filtration rate 12 months after kidney transplantation (104 KTRs, living donors excluded)

## Version 1 11/18/2021

In-Press Preview

## Version 2 01/11/2022

Electronic publication
