# Supplementary material for: Cell stress response impairs de novo NAD+ biosynthesis in the kidney
Source: JCI Insight. 2022 Jan 11;7(1):e153019. doi: 10.1172/jci.insight.153019 (PMC8765040; doi:10.1172/jci.insight.153019)
Supplement: Supplemental data [file jciinsight-7-153019-s040.pdf]

SUPPLEMENTARY FIGURE 1

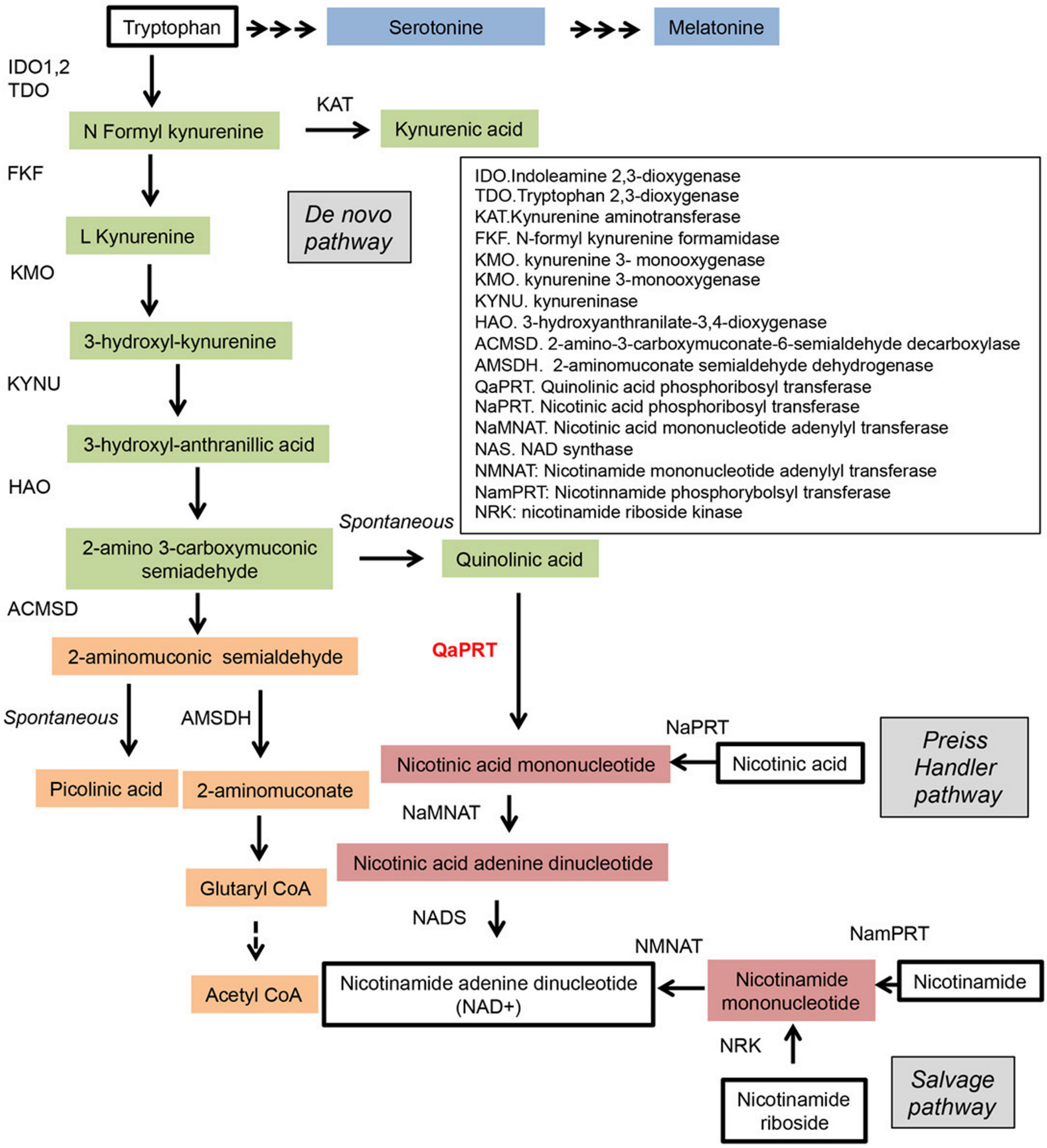

SUPPLEMENTARY FIGURE 2

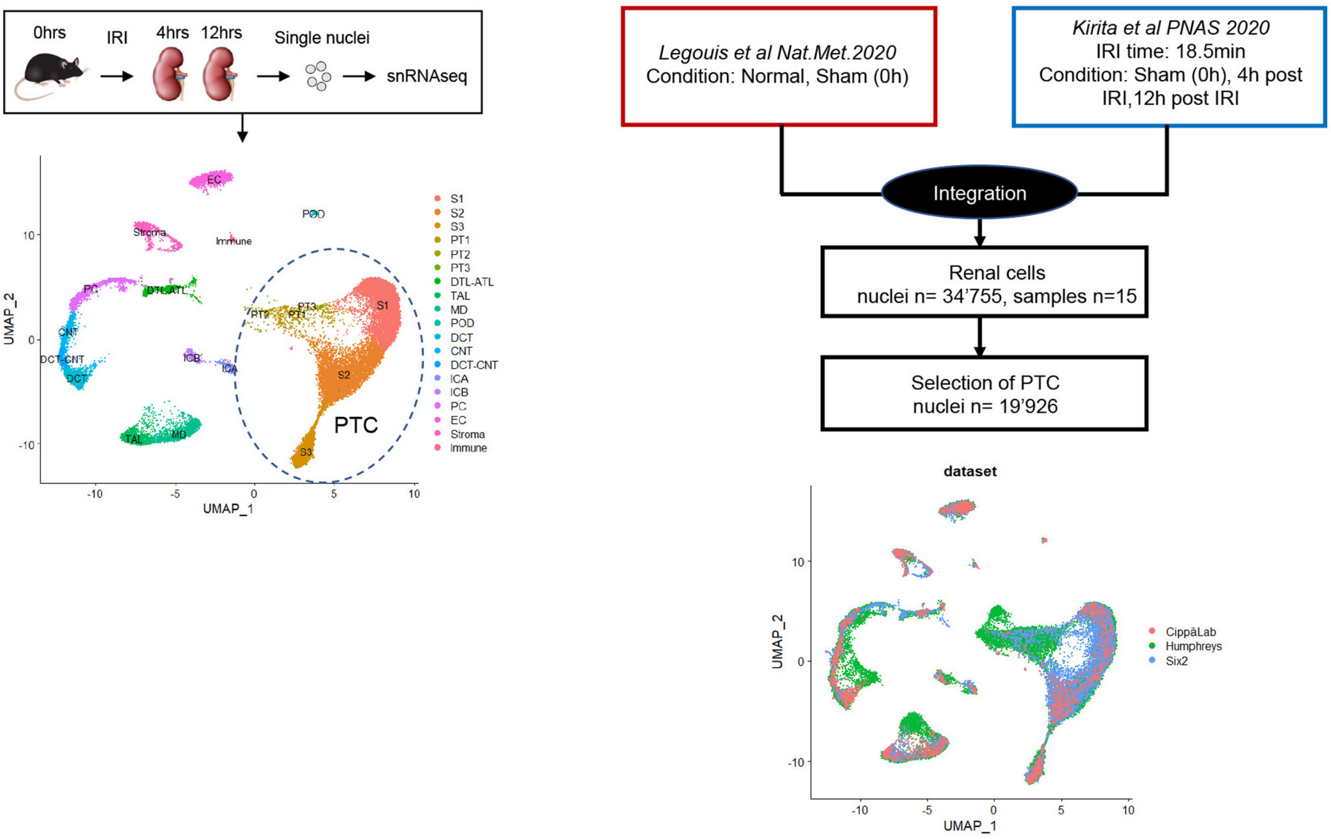

SUPPLEMENTARY FIGURE 3

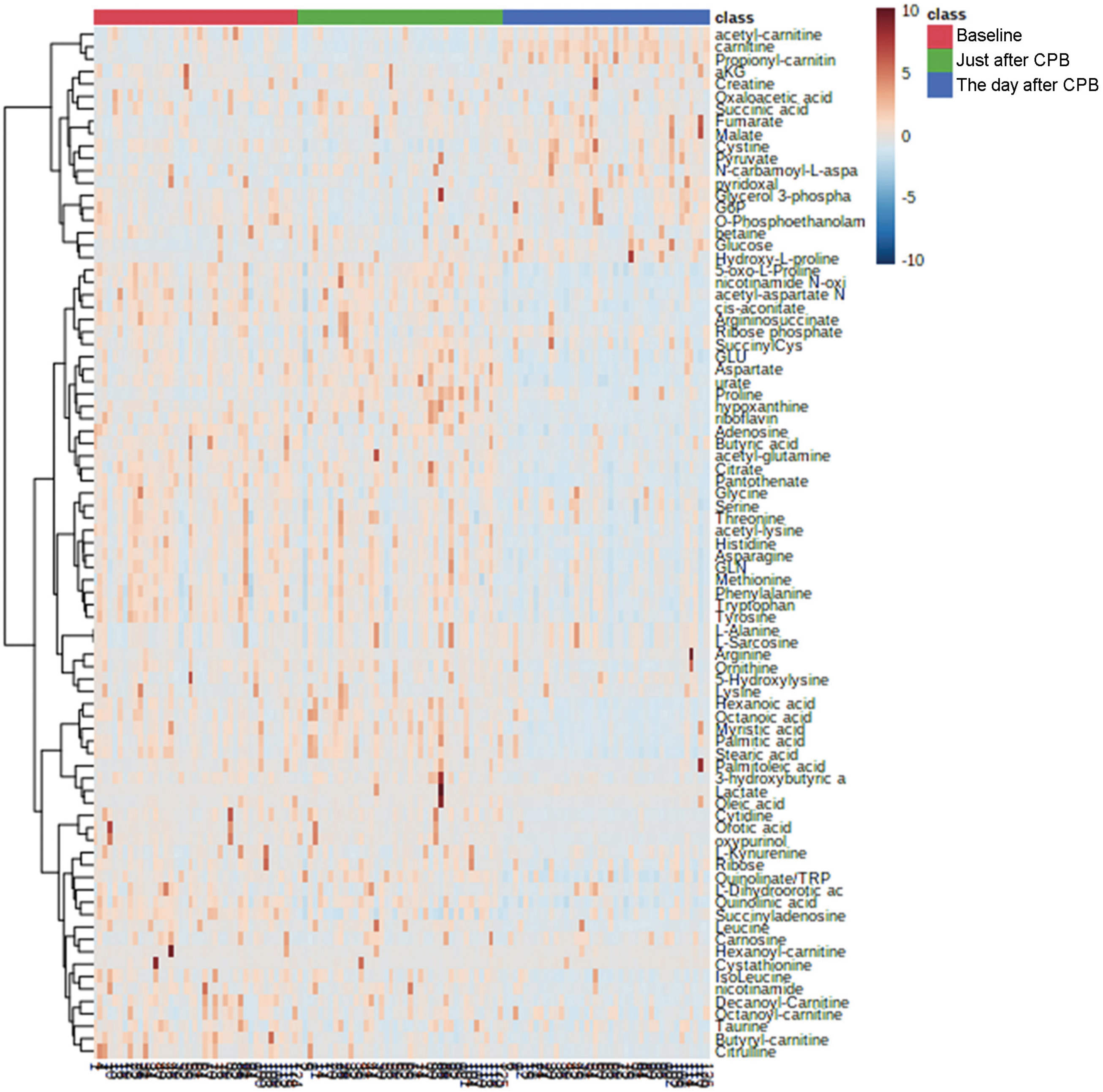

SUPPLEMENTARY FIGURE 4

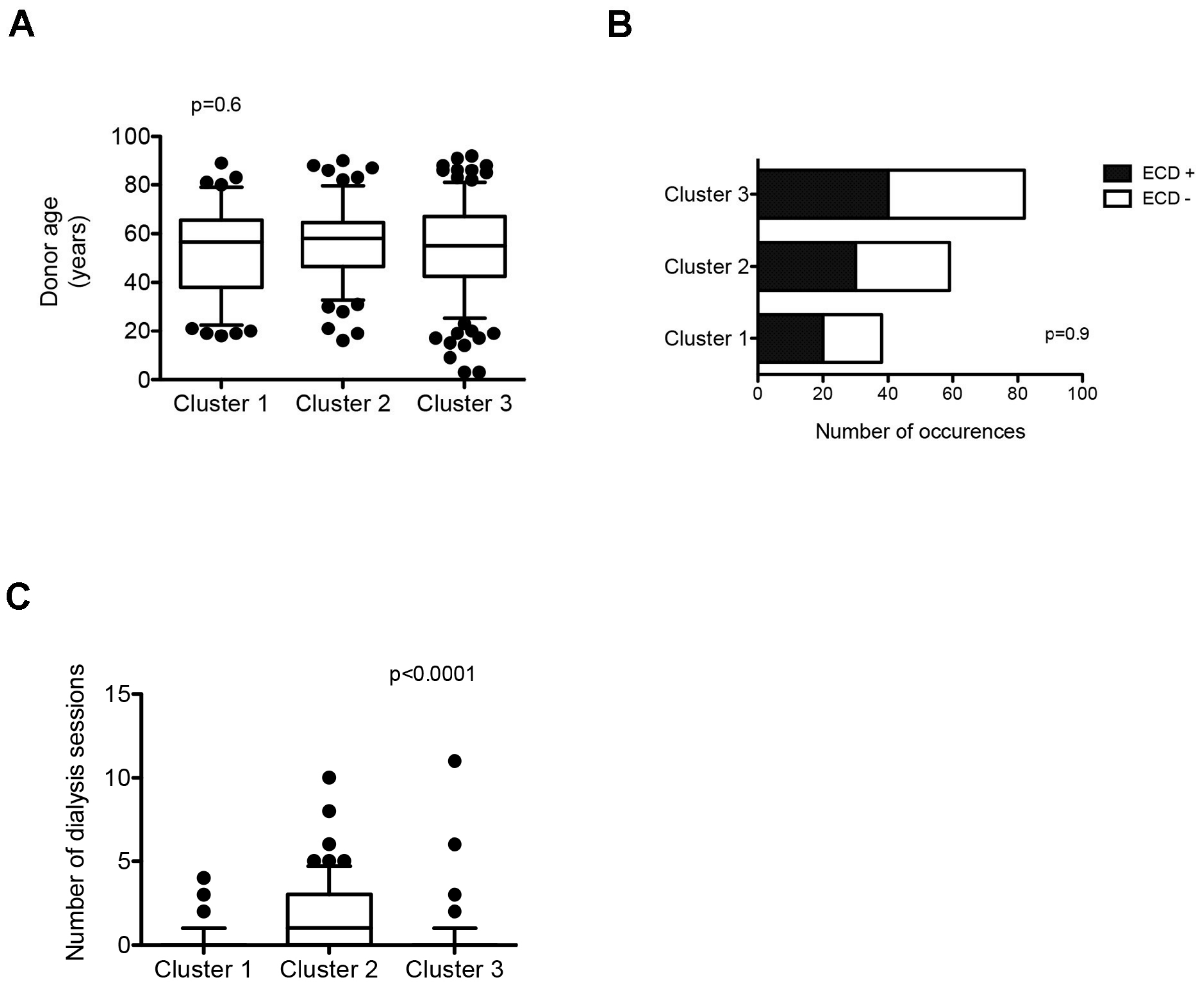

SUPPLEMENTARY FIGURE 5

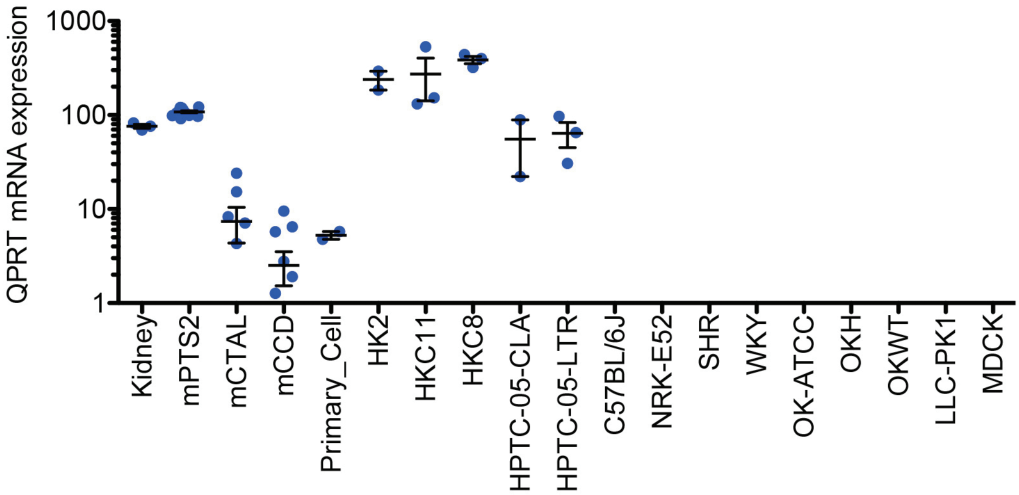

SUPPLEMENTARY FIGURE 6

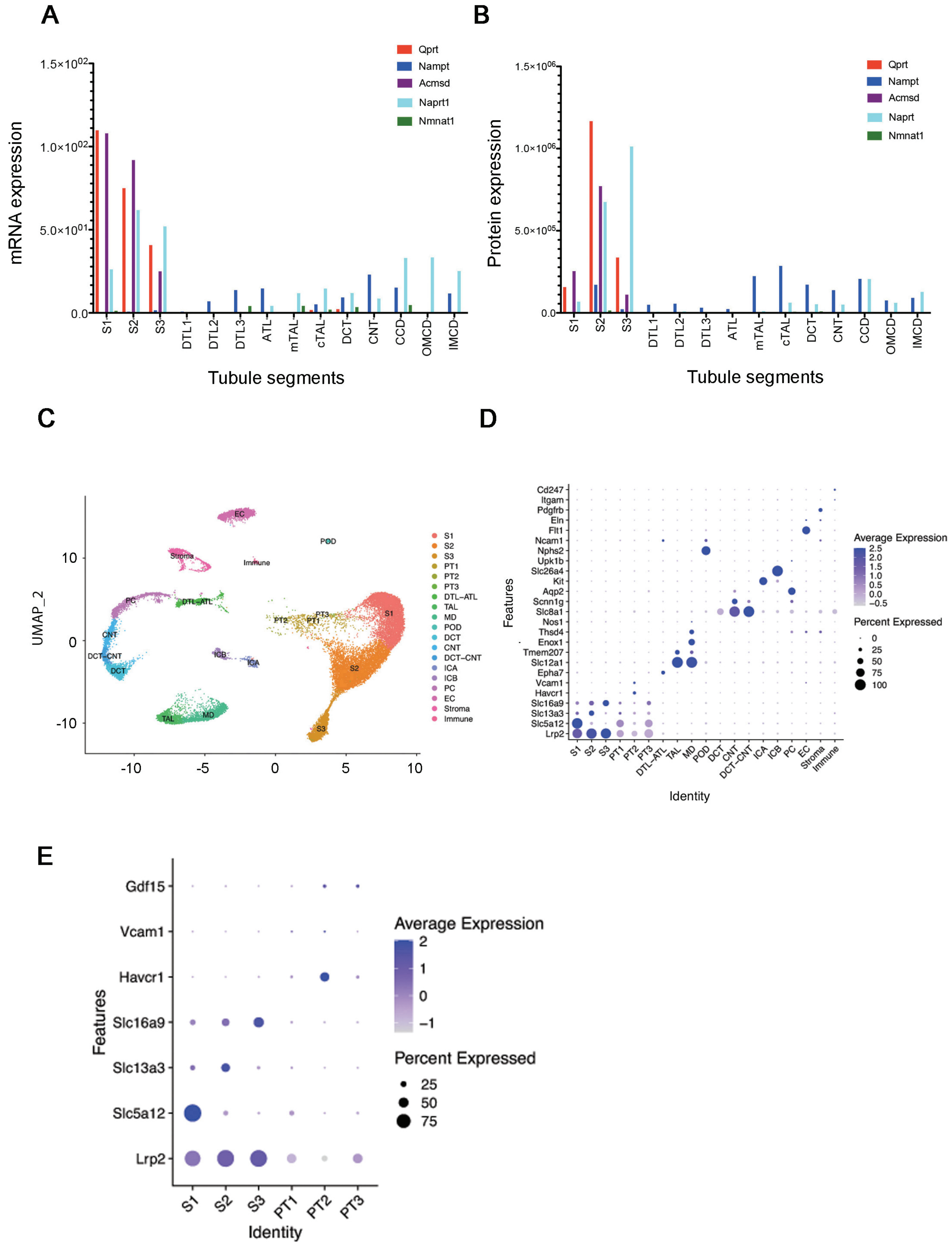

SUPPLEMENTARY FIGURE 7

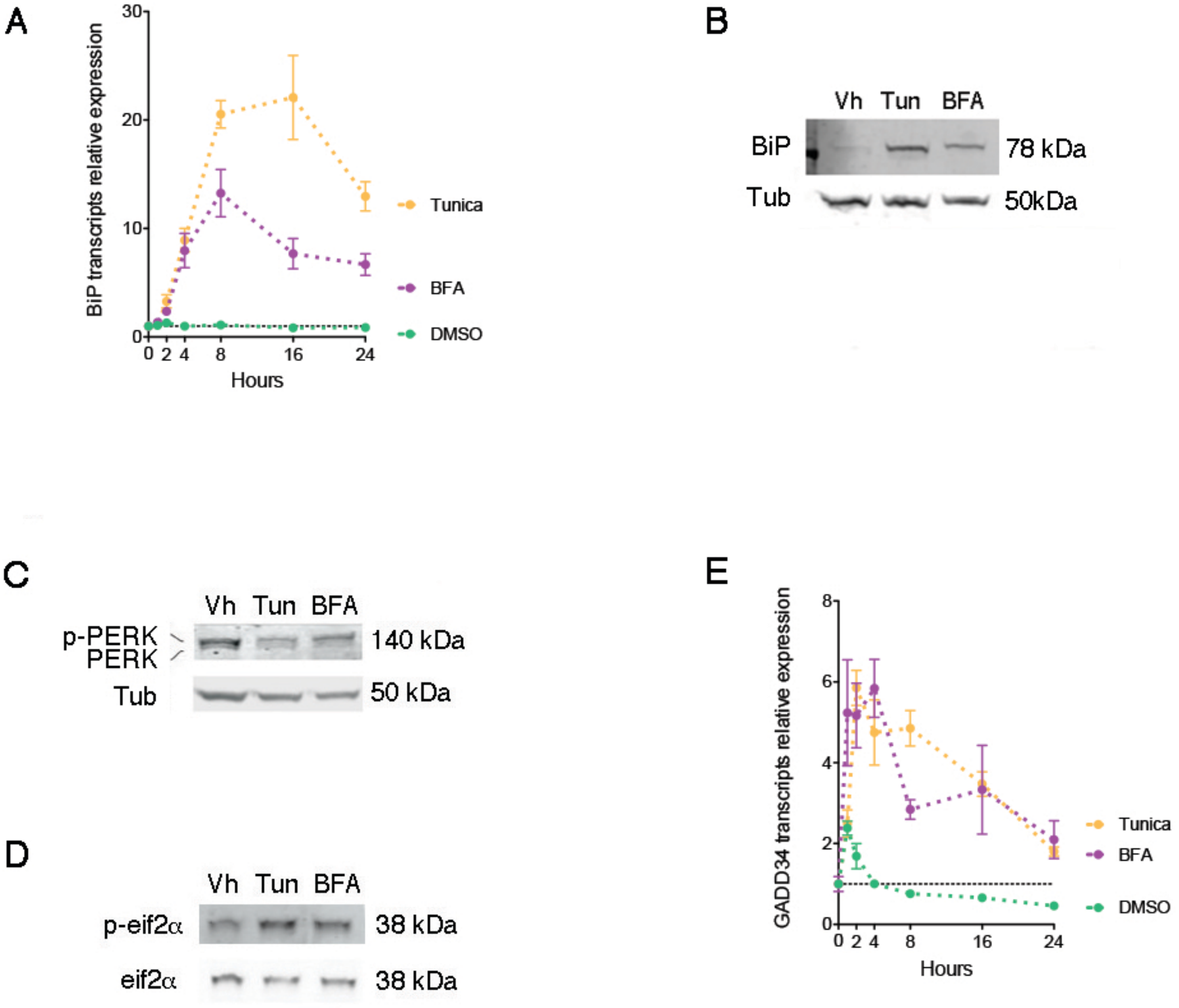

SUPPLEMENTARY FIGURE 8

A

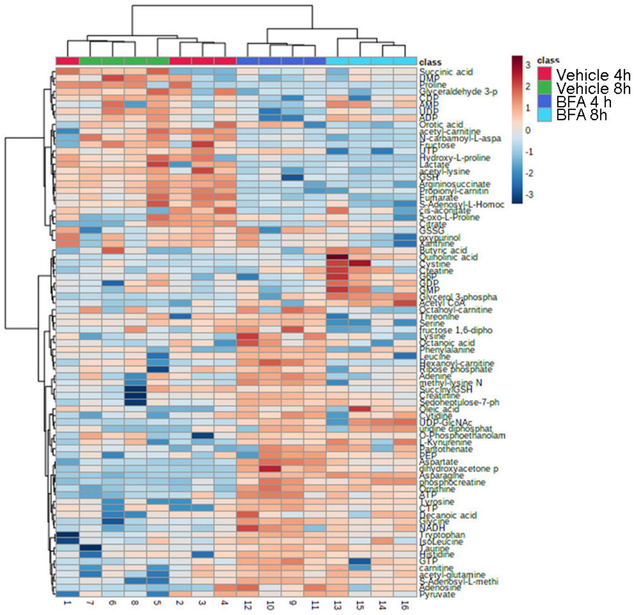

B

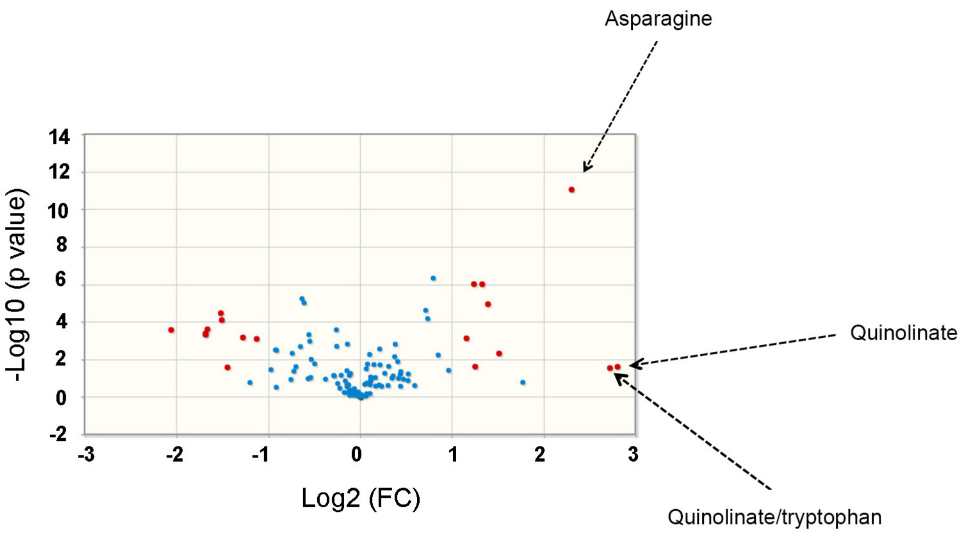

Brefeldin A vs vehicle

SUPPLEMENTARY FIGURE 9

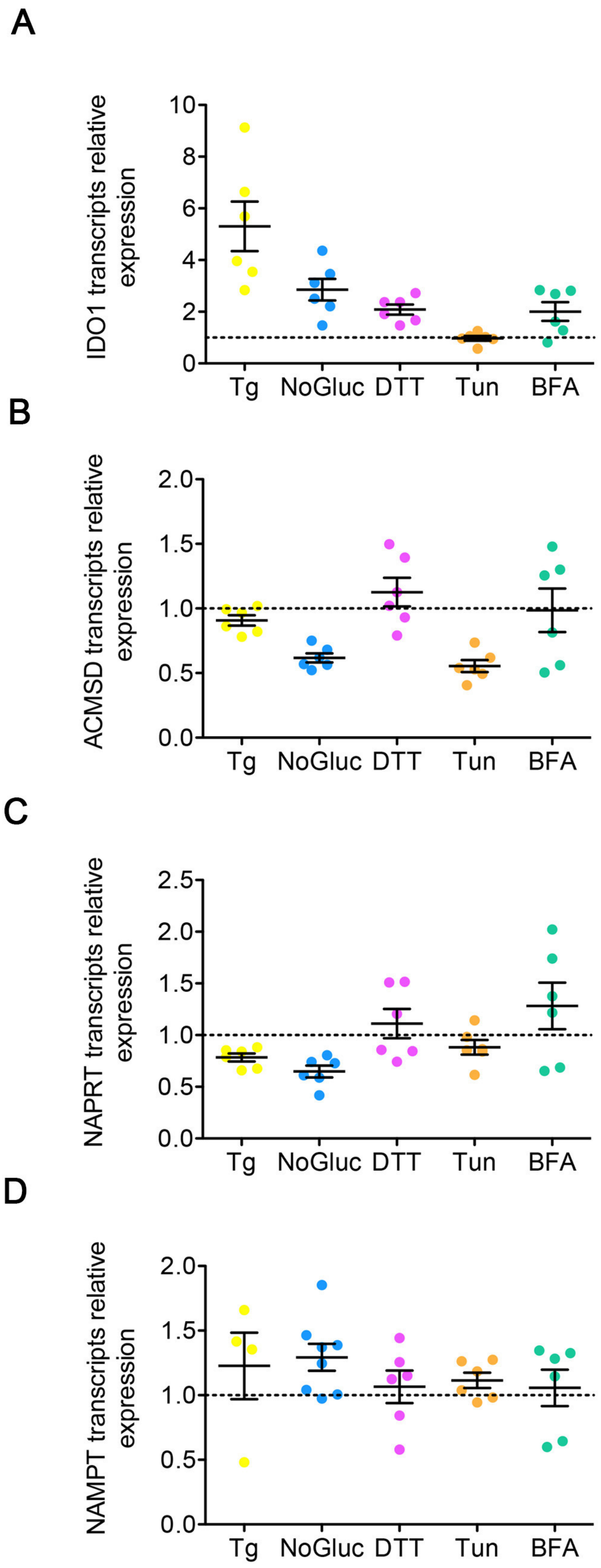

SUPPLEMENTARY FIGURE 10

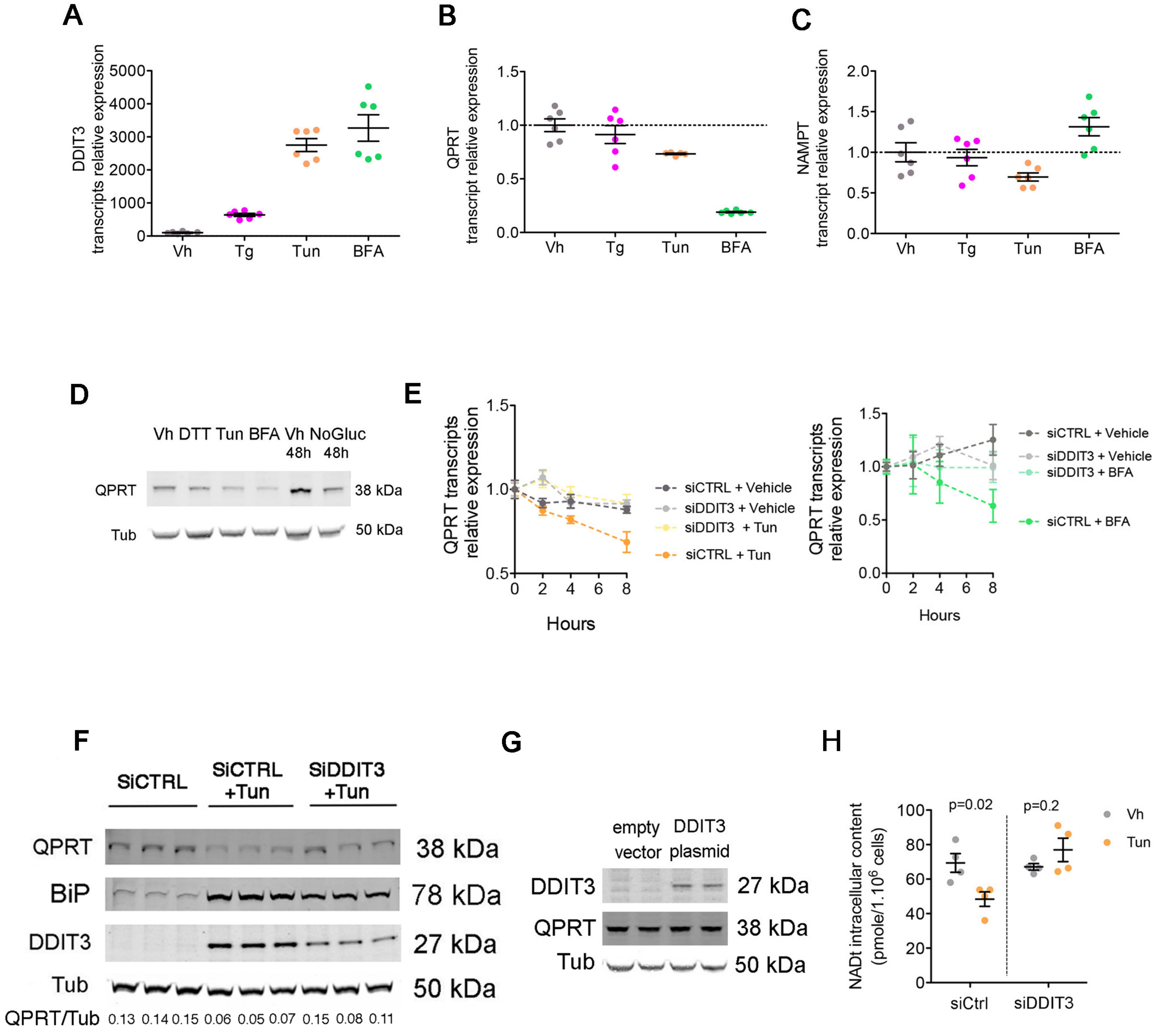

SUPPLEMENTARY FIGURES LEGENDS

SUPPLEMENTARY FIGURE 1

Representation of the tryptophan metabolic pathways

SUPPLEMENTARY FIGURE 2

Schematic of the experimental strategy for snRNAseq

SUPPLEMENTARY FIGURE 3.

Hierarchical clustering (Ward method) of urine samples of 41 patients collected at baseline, just after cardiopulmonary bypass (CPB) and the day after CPB. Each column represents the relative composition of metabolites identified by liquid chromatography coupled with tandem mass spectrometry using SeQuant ZIC-pHilic columns. Each line represents a patient. Samples were normalized by median and data were autoscaled. Each metabolite level is reported to urine creatinine.

SUPPLEMENTARY FIGURE 4.

A. Box and whiskers plots representing donor's age in groups of patients identified by hierarchical clustering of the metabolome of 237 urine samples collected 10 days after kidney transplantation. The ends of the box are the upper and lower quartiles, the median is marked by a vertical line inside the box, and the whiskers are the two lines outside the box that extend to the highest and lowest observations. p value was computed using one-way ANOVA.
B. Histograms representing the proportion of Extended Criteria Donors (ECD) in groups of patients identified by hierarchical clustering of the metabolome of 237 urine samples collected 10 days after kidney transplantation. p value was computed using one-way ANOVA.
C. Box and whiskers plots representing the number of dialysis sessions after kidney transplantation in groups of patients identified by hierarchical clustering of the metabolome of 237 urine samples collected 10 days after kidney transplantation. The ends of the box are the upper and lower quartiles, the median is marked by a vertical line inside the box, and the whiskers are the two lines outside the box that extend to the highest and lowest observations. p value was computed using one-way ANOVA.

SUPPLEMENTARY FIGURE 5.

Histograms representing the expression of QPRT transcripts in various human and non-human cell lines. Data are from public repositories pertaining to the transcriptome of these cells lines (https://esbl.nhlbi.nih.gov/JBrowse/KCT/)

SUPPLEMENTARY FIGURE 6.

A. Histograms representing the expression of Qprt, Nicotinamide phosphoribosyl transferase (Nampt), aminomuconate semiialdheyde decarboxylase (Acmsd), nicotinic acid phopshoribosyl transferase (Naprt)1, nicotinamidedinucleotide adenylyl transferase (Nmnat)1 transcripts in rat tubules. Data are from public repositories pertaining to the transcriptome (NCBI accession GSE56743 and the publicly accessible webpage (https://helixweb.nih.gov/ESBL/Database/NephronRNAseq/index.html)
B. Histograms representing the expression of Qprt, Nampt, Acmsd, Naprt1, Nmnat1 proteins in rat tubules. Data are from public repositories pertaining to the proteome (NCBI accession PDX016958 and the data publicly available online at the Kidney Tubule Expression Atlas website (https://esbl.nhlbi.nih.gov/KTEA/).
C. Uniform manifold approximation and projection (UMAP) plot of control kidney samples analyzed by single nucleus RNA sequencing (snRNAseq) (n=8; 25,896 cells) identify the different cellular components of the kidney. Proximal Tubule Segment S1, S2, S3; new (injured) proximal tubule clusters (PT1, PT2, PT3); DTL, descending limb of loop of Henle; ATL, thin ascending limb of loop of Henle; TAL, thick ascending limb of loop of Henle; POD, podocytes; MD, macula densa; DCT, distal convoluted tubule; CNT, connecting tubule; ICA,

type A intercalated cells of collecting duct; ICB, type B intercalated cells of collecting duct; PC, principle cells; EC, endothelial cells.

D. Dot plot displaying gene expression for the renal cell types of control kidney samples analyzed by single nucleus RNA sequencing (snRNAseq) (n=8; 25,896 cells).

E. Dot plot displaying differentiation and injury genes expression in proximal tubule segments (S1, S2, S3), and new (injured) proximal tubule clusters (PT1, PT2, PT3) of 7 mouse IRI and 8 control kidney samples analyzed by single nucleus RNA sequencing (snRNAseq) (n=19,926 cells) (n= 19,926 cells).

SUPPLEMENTARY FIGURE 7.

A. Time course analysis of the relative expression of BiP transcripts measured by RT-qPCR in HK2 cells incubated with either with vehicle (DMSO), 2.5 μg/ml tunicamycin (Tun), 5 μg/mL brefeldin A (BFA), or with for up to 24 h (4 replicates). Bars represent mean±sem.

B. Immunoblot representing BiP and tubulin protein expression in HK2 cells 24 hours after incubation with DMSO (vehicle), 2.5 μg/ml tunicamycin (Tun), or 5 μg/mL brefeldin A (BFA).

C. Immunoblot representing PERK and tubulin protein expression in HK2 cells 24 hours after incubation with DMSO (vehicle), 2.5 μg/ml tunicamycin (Tun), or 5 μg/mL brefeldin A (BFA).

D. Immunoblot representing eif2α, phospho-eiF2α, and tubulin protein expression in HK2 cells 24 hours after incubation with DMSO (vehicle), 2.5 μg/ml tunicamycin (Tun), or 5 μg/mL brefeldin A (BFA).

E. Time course analysis of the relative expression of GADD34 transcripts measured by RT-qPCR in HK2 cells incubated with either with vehicle (DMSO), 2.5 μg/ml tunicamycin (Tun), 5 μg/mL brefeldin A (BFA) for up to 24 h (4 replicates). Bars represent mean±sem.

SUPPLEMENTARY FIGURE 8.

A. Hierarchical clustering (Ward method) of HK2 cells incubated with 5 μg/ml BFA or dimethylsulfoxide (DMSO) for 4 h and 8 h (n=4 per condition). Each column represents the relative composition of metabolites identified by liquid chromatography coupled with tandem mass spectrometry using SeQuant ZIC-pHilic columns. Each line represents a condition or a replicate. Samples were normalized by sum and data were autoscaled.

B. Volcano plot comparing urinary metabolites of HK2 cells incubated with or without 5 μg/ml brefeldin A (BFA) for 8 hours. The x-axis is Log2 [fold change for BFA/vehicle] and y-axis is - Log10 (p.value) based on p values computed using a two-sided unpaired t-test without adjustment for multiple comparisons. Red points indicate metabolites that are above the threshold for the twofold abundance with a p value <0.05.

SUPPLEMENTARY FIGURE 9.

Scatter dot plots representing the relative expression of Indoleamine-pyrrole 2,3-dioxygenase (IDO1), Aminocarboxymuconate Semialdehyde Decarboxylase (ACMSD), Nicotinate Phosphoribosyltransferase (NAPRT), and (Nicotinamide phosphoribosyltransferase (NAMPT) transcripts measured by RT-qPCR in HK2 cells incubated 8 hours with either with 0.25 mM thapsigargin (Tg), with 1 mM dithiotreitol (DTT), with 2.5 μg/ml tunicamycin (Tun), with 5 mg/mL brefeldin A (BFA), or 48 hours in a glucose-deprived culture medium (No Gluc.), and compared with vehicle(DMSO)-treated cells. The data were obtained from 3 to 7 independent experiments. Bars represent mean±sem.

SUPPLEMENTARY FIGURE 10.

A, B, C. Scatter dot plots representing the relative expression of QPRT (A), DDIT3 (B) and NAMPT (C) transcripts measured by RT-qPCR in primary cultured proximal tubular cells incubated 8 hours with either with 0.25 μM thapsigargin (Tg), with 2.5 μg/ml tunicamycin (Tun), with 5 μg/mL brefeldin A (BFA), and compared with vehicle-treated cells. Bars represent mean±sem.

D. Immunoblot representing QPRT, DDIT3 and tubulin protein expression in primary cultured proximal tubular cells 24 hours after incubation with DMSO (vehicle), 1 μM dithiotreitol (DTT),

2.5 μg/ml Tun, with 5 μg/mL BFA, or 48 hours in a glucose-deprived culture medium (No Gluc.). The immunoblot shown is representative of 3 independent experiments.

E. Time course analysis of the relative expression of QPRT transcripts measured by RT-qPCR in primary cultured PTCs transfected with DDIT3 siRNA (siDDIT3) or with control siRNA (siScramble) and incubated 8 hours with either 2.5 μg/ml tunicamycin (Tun) (Left), 5 μg/mL brefeldin A (BFA) (right), and compared with vehicle-treated cells. Bars represent mean±sem

F. Immunoblot representing the expression of QPRT, BiP, DDIT3, and Tubulin proteins in primary cultured PTCs transfected with DDIT3 siRNA (siDDIT3) or with control siRNA (siScramble) and incubated with 2.5 μg/ml tunicamycin (Tun), or with DMSO (vehicle) for 24h. The immunoblot shown is representative of two independent experiments.

G Immunoblot representing the expression of QPRT and tubulin proteins in primary cultured PTCs transfected with a pcDNA3.1 vector expressing DDIT3 or an empty vector for 24h. The immunoblot shown is representative of two independent experiments.

H. Scatter dot plots representing the concentrations of total NAD concentrations in primary cultured PTCs transfected with DDIT3 siRNA (siDDIT3) or with control siRNA (siScramble) and incubated with 2.5 μg/ml tunicamycin (Tun) or DMSO (vehicle) for 24h. p value was computed with a Student's t-test.

Supplementary table 1. RT-qPCR primers list

Human-expression
huBIP-FwdGGTGAAAGACCCCTGACAAA,
huBIP-RevGTCAGGCGATTCTGGTCATT;
huNAMPT-FwdGTGACTTAAGCAACGGAGCG
huNAMPT-RevGGAGGATGTTGAACTCGGCT;
huQPRT-FwdGAGGGAAAGGAAGGATGGAAAG;
huQPRT-RevTGGGTGCATGAAGGATGAAG ;
huDDIT3-Fwd TGGAAGCCTGGTATGAGGAC
huDDIT3-Rev TGTGACCTCTGCTGGTTCTG
huCD38-Fwd ATGTTCACCCTGGAGGACACGCTGCT
huCD38-Rev CTCAGGATTTTTCACACACTGAAG
huPGC-1a-FwdCCAAAGGATGCGCTCTCGTTCA;
huPGC-1a-RevCGGTGTCTGTAGTGGCTTGACT
huNAPRT-FwdTCCCCAACTTCCTAGCAGTC
huNAPRT-RevGAAGACCTTGCGGATCTCCT
huACMSD-FwdCAGGAGCTCTTTCCTGTCTATG
huACMSD-RevGCCAAGGGAGCCAGTATTT
huIDO1-FwdGTGAAAGCTCTGGTCTCCCT
huIDO1-RevTCAGTGCCTCCAGTTCCTTT
huRPL13A-FwdCCTGGAGGAGAAGAGGAAAGAGA
huRPL13A-RevGAGGACCTCTGTGTATTTGTCAA
huIGADD34-FwdAGGAGGCTGAAGACAGTGGA
huIGADD34-RevGGCCATCTGCAAATTGACTT

Mouse
mQprt-FwdGTTCTGTGGGACACACATTGA
mQprt-Rev GGGTATCTCCTTCAGCAAACAG
mDdit3-FwdCAACAGAGGTCACACGCACA
mDdit3-RevGGCACTGACCACTCTGTTTC

Supplementary table 2. Description of the cohort of 41 patients undergoing cardio-pulmonary bypass

**Characteristic**	**All** (n=41)	**AKI** (n=11)	**No AKI** (n=30)	**p value**
Age (years)	62±15	64.5±4.5	61.5±3	0.5
Men, n (%)	25 (60)	6 (54)	20 (66)	0.31
BMI (kg/cm^2)	26±5	27±0.4	25±1	0.31
Preexisting medical condition, n (%)				
• Hypertension	20 (49)	7 (63)	14 (46)	0.5
• Diabetes	2 (4.7)	1 (1)	1 (8)	0.5
• Obesity	7 (16)	2 (18)	5 (16)	1
• Atherosclerotic disease	6 (14.2)	2 (18)	4 (16)	0.8
• CKD	9 (21)	4 (36)	5 (16)	0.3
Preoperative characteristics				
• eGFR (ml/min/1.73 m^2)	74±23	63±6	78.5±4	0.5
• Ejection fraction (%)	60±9.5	62±3	61.1±1.5	0.6
• Euroscore 2$	2.5±3	3±0.5	2.5±0.5	0.5
Preoperative medications, n (%)				
• RAS inhibitors	17 (44)	4 (33)	14 (46)	0.4
• Diuretics	12 (28)	6 (23)	5 (41)	0.3
• Beta blockers	22 (52)	8 (66)	14 (46)	0.25
Indications, n (%)				
• CABG	10 (24)	5 (41)	6 (20)	0.15
• Mitral valve	8 (19)	6 (20)	2 (16)	0.8
• Aortic valve	24 (57)	8 (66)	16 (66)	0.45
• Ascending aorta	6 (14)	1 (8)	5 (16)	0.45
• Others	6 (14)	0	5 (16)	0.45
Operative parameters				
• CBP length (min)	99±23	110±10	95±6.5	0.25
• Aortic cross-clamp length (min)	75±31	80±9	71±5.5	0.25
• Whole procedure length (min)	296±45	309±12	287±8	0.1
• Fluid loading (ml)	1570±710	1240±201	1686±125	0.07
• Blood loss (ml)	3020±1382	3270±422	2908±268	0.45
• Cell Saver (ml)	795±625	693±180	843±120	0.5
• Per CBP urine output (ml/kg/h)	61±86	65±25	61±16	0.9
• Per procedure urine output (ml/kg/h)	142±106	162±31	138±20	0.5
Post-operative parameters				
• 24 hour fluids administration (ml)¶	2002±737	2145±210	1950±135	0.5
• Catecholamine use (days)§	0.5±1.1	1±0.5	0.5±1	0.05
• Dialysis, n (%)	3 (7)	3 (25)	0	0.004
• ICU stay (days)	3±3	5±1	2.8±0.6	0.04
• Hospital stay (days)	15±8	19±9	14±1.5	0.04
• Death, n (%)	2 (5)	2 (16)	0 (0)	0.02

Plus-minus values are means±standard error.

BMI denotes body mass index; DSA, donor-specific antibodies; CKD, chronic kidney disease; eGFR, estimated glomerular filtration rate; RAS, renin-angiotensin system; CABG, coronary artery bypass grafting; CPB, cardiopulmonary bypass; ICU, intensive care unit.

$ Euroscore 2, www.euroscore.org/calc.html
¶ Includes albumin, Ringer's lactate, plasmion, or G5%.
§ Includes norepinephrine, dobutamine or adrenaline.

AKI is defined according to the AKI-KDIGO definition

Supplementary tables 3. Parameters associated with uQ/T

Supplementary table 3A. Parameters associated with uQ/T the day after cardiopulmonary bypass

Characteristic	Estimate	p value
Sex (female)	-3.1	0.02
BMI (kg/m^2)	-1.9	0.5
Age (years)	15.5	0.07
eGFR at baseline (ml/min/1.73 m^2)	-36.5	0.004
Left ventricular ejection fraction (%)	-4	0.4
CPB duration (minutes)	23.5	0.15
Aortic clamp time (minutes)	23.7	0.1
Procedure length (minutes)	30	0.2
Noradrenaline use	41	0.7
Intraoperative volume expansion (ml)	235	0.5
Intraoperative blood loss (ml)	773	0.2
Intaroperative time spent with MAP <60 mmHg (%)	0.04	0.2
Coronary bypass	1.8	0.25
Mitral valve surgery	-2.4	0.07
Aortic valve surgery	-0.3	0.7
Aortic surgery	0.5	0.7

Supplementary table 3B. Multivariate analysis of parameters associated with uQ/T the day after cardio pulmonary bypass.

Characteristic	Estimate	95% CI	p value
Sex (female)	-0.1	-0.17 to -0.02	0.01
Age (years)	0.003	-0.002 to 0.008	0.25
eGFR baseline (ml/min/1.73 m^2)	0.004	-0.007 to -0.0006	0.02
Aortic clamp duration (minutes)	0.001	-0.0008 to 0.004	0.24
Mitral valve surgery	-0.06	-0.04 to 0.1	0.4

Model F ratio 4.85; p=0.0018

BMI denotes body mas index
eGFR denotes estimated glomerular filtration rate (CKD-EPI formula)
MAP denotes mean arterial pressure

Supplementary tables 4. Multivariate analysis of parameters associated with AKI occurrence in the week after cardio pulmonary bypass.

Supplementary table 4A. Model including parameters associated with AKI at day 1 in the suppl. table 2
Model Chi2: 13.8 p=0.007

**Characteristic**	**Estimate**	**95% CI**	**p value**
Whole procedure length (min)	-0.01	-0.03 to 0.008	0.27
Fluid loading (ml)	0.001	-0.00006 to 0.003	0.06
Catecholamine use (days)	0.003	-0.002 to -0.01	0.4
uQ/T at day 1	-4	-9 to -1.4	0.003

Supplementary table 4B. Model including parameters associated with uQ/T at day 1 in the suppl. table 3B
Model Chi2: 8.9; p=0.03

**Characteristic**	**Estimate**	**95% CI**	**p value**
Sex (female)	-0.15	-0.1 to 0.7	0.7
eGFR baseline (ml/min/1.73 m^2)	0.02	-0.01 to 0.06	0.3
uQ/T at day 1	-3.2	-7.4 to 0.006	0.049

Supplementary tables 5. Clinical characteristics of the cohort of 237 kidney transplant recipients

Supplementary table 5A. Demographic and clinical characteristics of the cohort of kidney transplant recipients who had a urine sample analysis 10 days after transplantation.

**Characteristic**	n=237
Age (yrs)	50.5±15.4
Male sex-n (%)	148 (62)
Cause of ESRD-n (%)	
• Primary GN	56 (24)
• Diabetes	28 (12)
• Cystic/hereditary	45 (19)
• Secondary GN	15 (6.5)
• Hypertension	14 (6)
• Interstitial nephritis	16 (7)
• Miscellaneous	12 (5)
• Uncertain	43 (18.5)
Donor age (yrs)	54 ±19
Living donor-n (%)	57 (24)
Extended criteria donor-n (%)	89 (49)
Retransplantation-n (%)	36 (15)
Preformed anti HLA DSA-n (%)	100 (42)
Cold ischemia time (hours)	16.4±10
Delayed graft function-n (%)	53 (23)
Serum creatinine at 10 days (μmol/L)	260±238

Plus-minus values are means±standard error.
ESRD denotes end stage renal disease, GN glomerulonephritis, DSA donor specific antibodies.
Extended criteria donors (ECD) are normally aged 60 years or older, or over 50 years with at least two of the following conditions: hypertension history, serum creatinine > 133 μmol/L or cause of death from cerebrovascular accident.

Supplementary table 5B. Initial immunosuppressive regimens of the cohort of kidney transplant recipients who had a urine sample analysis 10 days after transplantation.

**Characteristic**	n=237
Basiliximab-n (%)	94 (39)
ATG-n (%)	131 (55)
Tacrolimus-n (%)	188 (80)
Cyclosporine-n (%)	44 (18)
IMPDHi-n (%)	244 (100)
MTORi-n (%)	3 (8)
Steroids-n (%)	244 (100)
Anti CD20 immunotherapy-n (%)	29 (12)
Intravenous polyvalent immunoglobulins-n (%)	122 (51)

ATG denotes antithymocyte globulins, IMPDHi inhibitor of Inosine Monophosphate Deshydrogenase, MTORi inhibitor of Mechanistic Target of Rapamycin.

Supplementary table 6. Metabolites discriminating cluster 2 versus cluster1+3.
Among 237 kidney transplant recipients. 3 groups of patients (clusters) have been identified according to their urinary metabolome measured 10 days after transplantation.

Metabolite	Fold change	p value
Histidine	0.3	5.3e-14
Quinolinic acid	2.1	3.7e-10
Ribose	2.3	1.5e-09
Quinolinate/Tryptophan	6.7	1.5e-08
5-Hydroxylysine	0.4	3.9e-08
Aminoadipate	2.5	1.3e-06
Riboflavin	3.1	4.8e-05
Cystathionine	2.5	0.0001
Lysine	0.4	0.0002
Pyridoxal	2.1	0.0007
Pantothenate	0.4	0.02

Supplementary table 7. Demographic and clinical characteristics of a non-selected cohort of 55 patients with a chronic kidney disease (CKD).

**Characteristic**	n=55
Age (years)	65±17
Men, n (%)	39 (70)
BMI (kg/cm^2)	27±5
Hypertension, n (%)	42 (72)
Diabetes, n (%)	19 (34)
Kidney transplant, n (%)	19 (34)
Cause of kidney disease (in native kidneys)	
• Primary glomerulonephritis	8
• Amyloidosis	5
• AAN	4
• Chronic tubulointerstitial nephropathy	4
• ADPKD	3
• Malformative uropathy	3
• MGRS	2
• Diabetes	4
• Hypertensive	1
• HUS	1
• Alport syndrome	1
eGFR (ml/min/1.73 m^2)	41±27
Serum creatinine (μmol/l)	229±167
Proteinuria (g/mmol)	0.15±0.3
Albuminuria (mg/mmol)	0.55±18

Plus-minus values are means±standard error
BMI denotes body mass index; AAN: ANCA associated nephropathy; ADPKD: autosomal dominant polycystic kidney disease; MGRS: monoclonal gammopathy of renal significance; HUS: hemolytic and uremic syndrome; eGFR: estimated Glomerular filtration rate by the CKD-EPI formula.

Supplementary tables 8. Intracellular metabolites associated with ER stress in HK2 cells

Supplementary table 8A. Metabolites that best discriminate Tunicamycin-treated cells from vehicle treated cells**.** Cells were incubated 8 h with vehicle or Tunicamycin. In bold are shown metabolites that are common to brefeldin A treated cells.

**Metabolite**	**Fold change**	**p value**
**Quinolinic acid**	**6.5**	**0.06**
**Asparagine**	**6.2**	**2.9e-06**
Cystine	3.1	0.09
Tyrosine	2.7	0.01
Folate	2.4	0.002
S-Adenosyl-L-methionine	2.3	0.03
Acetyl-glutamine	2.2	0.04
**UDP-GlcNAc**	**2.1**	**4e-06**
Cytidine	2	0.08
Fumarate	0.4	0.02
**Glyceraldehyde 3-phosphate**	**0.3**	**0.004**
**L-Dihydroorotic acid**	**0.3**	**0.07**
Glucose	0.3	0.07
Propionyl-carnitine	0.3	0.002
**N-carbamoyl-L-aspartic acid**	**0.19**	**0.0004**
Orotic acid	0.15	7.8e-05
Proline	0.15	0.02

Supplementary table 8B. Metabolites that best discriminate brefeldin A-treated cells from vehicle treated cells. Cells were incubated 8 h with vehicle or brefeldin A. In bold are shown metabolites that are common to tunicamycin treated cells.

	**Fold change**	**p value**
**Quinolinic acid**	**6.9**	**0.02**
**Asparagine**	**4.9**	**9.1e-12**
Acetyl CoA	2.8	0.004
Adenosine	2.6	1.1e-05
Glycerol 3-phosphate	2.5	1e-06
NADH	2.3	0.02
**UDP-GlcNAc**	**2.3**	**9.8e-07**
Creatine	2.2	0.0007
Orotic acid	0.4	0.0002
**Glyceraldehyde 3-phosphate**	**0.4**	**0.0005**
Citrulline	0.3	0.0004
Acetyl-lysine	0.3	0.0002
Argininosuccinate	0.3	3.5e-05
**L-Dihydroorotic acid**	**0.3**	**8.1e-05**
Fructose	0.3	0.02
Hydroxy-L-proline	0.3	0.0007
**N-carbamoyl-L-aspartic acid**	**0.2**	**0.0008**
